# Predictive and Prognostic Protein Biomarkers in Epithelial Ovarian Cancer: Recommendation for Future Studies 

**DOI:** 10.3390/cancers2020913

**Published:** 2010-05-26

**Authors:** Cécile Le Page, David G. Huntsman, Diane M. Provencher, Anne-Marie Mes-Masson

**Affiliations:** 1 Centre de recherche du Centre hospitalier de l’Université de Montréal (CR/CHUM), Institut du cancer de Montréal, 1560 Sherbrooke Est, Montreal, H2L4M1, QC, Canada; E-Mails: cecilelepage@yahoo.ca (C.L.P.); diane.provencher.chum@ssss.gouv.qc.ca (D.M.P.); 2 Department of Pathology and Genetic Pathology Evaluation Centre of the Prostate Research Center, Department of Pathology and Laboratory Medicine, University of British Columbia, Vancouver General Hospital, Vancouver, Canada; E-Mail: dhuntsma@bccancer.bc.ca (D.G.H.); 3 Translational and Applied Genomics, BC Cancer Agency, Room 3427, 600 West 10th Avenue, Vancouver, V5Z 4E6, BC, Canada; 4 Département d’Obstétrique et Gynécologie, Clinique de Gynécologie Oncologie, Université de Montréal, 1560 Sherbrooke Est, Montreal, H2L4M1, QC, Canada; E-Mail: diane.provencher.chum@ssss.gouv.qc.ca; 5 Département de Medicine, Université de Montréal, 1560 Sherbrooke Est, Montreal, H2L4M1, QC, Canada

**Keywords:** gynecologic cancer, outcome, immunohistochemistry

## Abstract

Epithelial ovarian cancer is the most lethal gynecological malignancy. Due to its lack of symptoms, this disease is diagnosed at an advanced stage when the cancer has already spread to secondary sites. While initial rates of response to first treatment is >80%, the overall survival rate of patients is extremely low, mainly due to development of drug resistance. To date, there are no reliable clinical factors that can properly stratify patients for suitable chemotherapy strategies. Clinical parameters such as disease stage, tumor grade and residual disease, although helpful in the management of patients after their initial surgery to establish the first line of treatment, are not efficient enough. Accordingly, reliable markers that are independent and complementary to clinical parameters are needed for a better management of these patients. For several years, efforts to identify prognostic factors have focused on molecular markers, with a large number having been investigated. This review aims to present a summary of the recent advances in the identification of molecular biomarkers in ovarian cancer patient tissues, as well as an overview of the need and importance of molecular markers for personalized medicine in ovarian cancer.

## 1. Introduction

Although epithelial ovarian cancer (EOC)s could originate either from normal ovarian surface epithelium (NOSE) itself or from the crypts or inclusion cysts arising from this surface epithelium [1], recent evidence suggests that high grade serous ovarian cancer can arise from the fallopian tube [2]. Even though the etiology of most EOCs is unknown, about 10% have been attributed to known genetic factors such as BRCA1 and BRCA2, or true mutations in mismatch repair genes such as MLH1 and MSH2, which causes predisposition to the Lynch syndrome [3]. Ovarian cancer is surgically staged using the FIGO classification, which is based on the volume and extent of tumor spread. Stage I tumors are limited to one or both ovaries. Stage II tumors are associated with pelvic extension. Stage III tumors are characterized by spreading outside the pelvis into the abdominal cavity or true retroperiteonal lymph node, and stage IV tumors present with liver parenchymal metastasis and/or distant metastasis. EOC is also graded according to the degree of differentiation: borderline or low malignant potential (LMPs) represent minimal deviation from their benign counterpart while well differentiated tumors are grade I, moderately differentiated are grade II, and poorly differentiated are grade III carcinomas. As this three part grading system for carcinomas was not reproducible, it has been replaced by a two category system with low and high grade carcinomas.

When the two category grading system is combined with histotype, the emergent classification system includes: serous (low grade and high grade), clear cell endometrioid, mucinous, and other rare types such as Brenner or undifferentiated [4,5]. The frequency of these subtypes in North America are high grade serous (HGS) cancer (70%), clear cell carcinomas (12%), endometrioid (11%) and finally low grade serous (LGS) and/or mucinous carcinomas (<5%). Although all of these subtypes are distinct in terms of their association with precursor lesions, risk factors and mutation profiles, the high grade serous cancers feature near universal p53 mutations and genomic instability whereas low grade serous cancers, endometrioid carcinomas, mucinous carcinomas and clear cell carcinomas have relatively stable genomes and rare tp53 mutations. High grade serous tumors show particular differences in terms of their development, genetic alterations and prognosis. This has led to the classification of ovarian cancer into two types: type 1 tumors, which are low grade and slowly developing (endometrioid, mucinous and low grade serous tumors), and type 2 tumors, which rapidly progress (high grade serous). In addition, the association of biomarker expression with survival varies substantially between subtypes, and can easily be overlooked in whole cohort analyses. Although these data suggest substantial differences between subtypes, until recently ovarian carcinomas were typically approached as a monolithic entity by researchers and clinicians. This practice impeded progress in understanding the biology or improving the management of the less common ovarian carcinoma subtypes. To avoid this effect, each subtype within a cohort should be analyzed individually. Therefore, molecular classifiers of ovarian cancer are of high clinical relevance in the management of these cancer patients. 

Over the last 15 years, the Gynecologic Cancer InterGroup GCIC, a cooperative of 16 international groups interested in the treatment of gynecologic cancer, has made an international effort to coordinate research and practice in the treatment of ovarian cancer, including therapy and prognosis factors [6]. The standard treatment for ovarian cancer patients is cytoreductive surgery, during which adequate staging is performed. Clinically, the most important prognostic factor is the presence of residual disease after initial de-bulking surgery. 

This treatment is followed by a platinum-based combination of chemotherapy, although the disease will recur and inevitably become resistant to further chemotherapy (reviewed in [7]). Two platinum compounds are in common use, cisplatin and carboplatin, with slightly different spectra of activity [8]. Carboplatin in combination with taxane is often chosen as a treatment following surgery for most stages of ovarian cancer [9]. For select patients with stage III EOC, intraperitoneal chemotherapy has become one of the standard options, but due to toxicity and health resource issues this has not been universally adopted. Currently, patients, disease and health care resources are important parameters when choosing therapeutic options [10]. 

While more than 80% of the patients will initially respond to treatment, recurrence is common but is generally observed within variable time intervals. Patients recurring within six months of first line treatment are considered as resistant, while recurrence at 6–24 months is considered a moderate response and greater than 24 months is considered a good prognosis. The high rate of recurrence and mortality of this disease (>80%) underscores the need for a greater understanding of the molecular basis of the disease and the development of new clinical tools for the detection and management of ovarian cancer patients. 

Without doubt, the age of personalized medicine is at our doorstep. In concrete terms, personalized cancer care has several goals that impact our society and the care of patients. In particular, before cancer is detected it should be possible, based on genetic and environmental factors, to estimate an individual’s risk for developing a particular cancer. Here the alteration of modifiable risk factors, and the close monitoring of high-risk individuals, should be attainable goals and risk-reducing strategies. Once diagnosed, personalized medicine means matching each cancer patient to the most appropriate treatment. This not only results in superior medical care, by improving effectiveness while diminishing toxicities, but it also impacts directly on health economics and quality of life issues. Finally, future clinical research, coupled to companion translational research studies, should inevitably improve performance at an individual level. This review focuses on molecular markers associated with patient outcome and provides a summary of studies identifying potential biomarkers for EOC (refer to supplementary file). 

## 2. Protein Biomarkers Associated with Chemoresistance and Survival

More than a thousand publications have identified potential prognostic markers of epithelial ovarian cancer. However, most of these proposed markers have an uncertain clinical value, their independent prognostic significance is unclear and none are used clinically. In this review, we introduce and discuss the main groups of biomarkers identified by immunohistochemistry. 

### 2.1. Oncogenes and Tumor Suppressors

#### 2.1.1. Tumor Suppressor p53

Although the oncoproteins p53 and Her-2 are among the most investigated markers in ovarian cancer, they have still not shown reproducible results in different studies (reviewed in [11] and supplementary file). The p53 protein is the most studied tumor suppressor, and mutations in the p53 gene and subsequent gene product have been related to most cancer types. De Graeff *et al.* [11] recently determined a prognostic value of p53 in ovarian cancer through a meta-analysis of 62 previously published studies using a total of 9448 patients. The authors also included a quality study assessment when performing their meta-analysis in order to help in evaluating and better understand the discordance between studies. The p53 status was analysis by IHC or mutational analysis. Out of 62 studies, 25 reported an association with poor survival while four were associated with improved survival. When the meta-analysis was restricted to serous tumors only, there was a significant association with poor prognosis. Although, histotype heterogeneity and chemotherapeutic treatment were not taken into account, a meta-regression analysis showed that the FIGO stage may influence the outcome predictive value of p53 and the prognostic significance of p53 seems also to be more restricted to low stage tumors [11]. Some other studies suggest a correlation between p53 status and response to platinum-based chemotherapy while strong discrepancies are noticed in clinical studies with paclitaxel-based treatment (reviewed in [12,13] and supplementary file). The apparent inconsistencies are likely due to different antibodies utilized, chemotherapeutic regimen and heterogeneity of histosubtypes, which render the interpretation of all these studies difficult. 

#### 2.1.2. Wilms Tumor: WT1

The Wilms Tumor gene (WT1) product was initially defined as a tumor suppressor gene involved in the development of Wilm’s tumor, but today it is considered capable of performing oncogenic functions. In studies describing WT1 expression in ovarian cancer, there seem to be differences in expression patterns among different histological subtypes with higher expression in the serous subtype [14,15,16,17]. Conflicting reports show either no prognostic value, or a significant unfavorable prognosis associated with WT1, when all histological subtypes or all grades of serous tumors are analyzed [14,15,16,18,19,20], while WT1 becomes a favorable marker in a restricted high grade serous cohort of patients [16,21]. However, it is not an independent marker of survival in multivariate analysis. WT-1 is very useful as a diagnostic marker for tumors with serous differention and can be used to differentiate high-grade serous cancers from mixed carcinomas and all prognostic effects are likely due to association with serous tumors [16].

### 2.2. Proliferation Markers

#### 2.2.1. Ki67

Numerous markers of interest under investigation in this group, which include Ki67, show contradictory observations [22,23,24,25,26,27,28,29,30,31] (supplementary file). Ki67 is the most studied proliferation marker in cancer research. It is a nuclear protein expressed in cells during the G1, S, G2 and M cell cycle phases and is absent in quiescent cells (phase G0). Ki67 is overexpressed in malignant tissues compared to benign or borderline tissues [32]. This overexpression seems to be correlated with the serous subtype [16,33] although the difference is not significant in all studies [30,31,32]. In most reports, high expression of Ki67 is associated with a poor patient outcome, either shorter survival or shorter disease-free survival [28,31,32,33,34,35,36,37,38,39,40,41,42] although not in all cases [16,30,43,44]. Interestingly, Kobel *et al.* [16] observed a significant association of Ki67 in a cohort of patients representing several histosubtypes of the disease but no association was observed when each subtype was individually analyzed. This result may explain some level of discrepancies between studies. However, in other studies including only serous patients an association between Ki67 and outcome was still observed [26,38,42] while in studies including several histological subtypes no association was observed [30,45] suggesting that other variables influence the predictive value of Ki67. Such variables may be related to treatment regimens which often varies between studies. 

#### 2.2.2. Proliferation Cell Nuclear Antigen or PCNA

PCNA is a protein cofactor of DNA polymerase during DNA replication. Ki67 is considered to be a more indicative proliferation marker than PCNA, and consequently PCNA is less frequently used. Using the monoclonal PC-10 antibody from DAKO Inc., no correlation has been observed between PCNA nuclear expression and survival of ovarian cancer patients [28,46] (Refere to Supplementary file). However some investigators showed a poor outcome in patients with highly proliferative ovarian cancer tissues [12,47,48] while other authors, analyzing a cohort of 92 patients, reported a better five-year survival in tissues with higher PCNA staining [49]. Interestingly, some investigators that have analyzed both Ki67 and PCNA in the same cohort of patients showed a concordant result with both markers [28,46,47]. This suggests that the significance of proliferation markers and outcome of patients is more dependent on the selected patient cohort than the proliferation marker chosen. In the same line of evidence, Nijman *et al.* [50] reported the influence of platinum-based chemotherapy on the increased expression of molecular markers such as p53 and PCNA. 

#### 2.2.3. Topoisomerases

Another set of proteins involved in cellular proliferation are topoisomerases. Topoisomerases are enzymes that alter the topologic states of DNA. In particular, Topoisomerase II (Topo II) catalyzes the relaxation of supercoiled DNA during DNA replication. Topo II is a marker of proliferation, expressed during the G1, S, G2 and M phases of cell cycle but absent during the resting Go phase. In addition, Topo II is also the molecular target of the chemotherapeutic agent etoposide and in cells treated with etoposide DNA breaks accumulate and trigger cell death. Topo II has been described as a marker for platinum-based chemotherapy sensitivity and is associated with shorter survival [39,51,52,53] (Refer to Supplementary file). It remained an independant variable in a multivariate analyses of survival [39,54]. However, it has not been described as a marker of platinum chemotherapy sensitivity in every study [54,55,56]. The discrepancy is likely due to differences in study design. 

### 2.3. Cell Cycle Regulators

**Figure 1:**
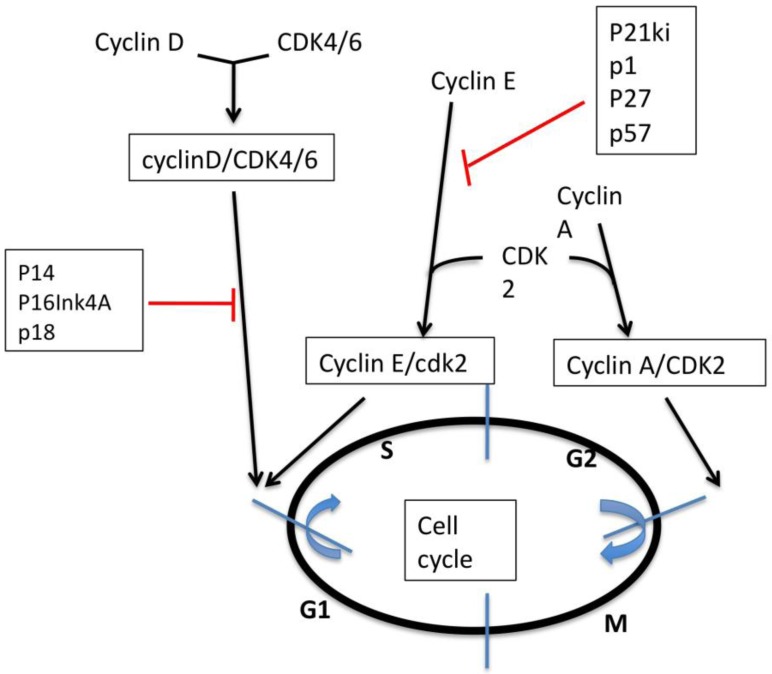
Brief schematic representation of cell cycle regulation.

There is strong evidence in the literature that cell cycle regulators are involved in ovarian cancer progression and can modify response to treatment. Cyclins are a family of proteins that control cell cycle progression by activating cyclin-dependent kinase (Cdk) enzymes (Figure 1). The cyclin-Cdk complexes are, in turn, regulated by kinase inhibitors, termed CKIs. Cyclins are expressed and then disappear at specific time points during the cell cycle. CyclinD/CDK4/6 and CyclinE/CDK2 regulate the transition from G1 to S phase of the cell cycle. They mediate the phosphorylation and inactivation of Rb, thus permitting cell cycle progression. Cyclin A interacts with Cdk2 at the late GI and S phase and form a complex essential for the entry in mitosis. The cell cycle is then regulated by two major families of CKIs. The INK4 family, which includes p14, p16^Ink4A^ and p18, inhibits Cyclin D/CDKs. The Cip/kip family, which includes p21^waf1^, p27^kip1^ and p57, inhibits Cyclin E/CDK. Cell cycle deregulation leading to oncogenesis can be caused by aberrant expression of positive regulators (cyclins) or loss of negative regulators (CKIs).

#### 2.3.1. Cyclins

**Cyclin E** is the most studied cyclin in EOC and is associated with poor prognosis in patients with serous EOC and in cohorts with a large majority of the serous subtype [57,58,59,60,61,62] (reviewed in [63] and Supplementary file), but not in non-serous cohorts [57] or tissue sets from patients with a minority of serous disease [41]. Surprisingly, Bedrosian *et al.* [62] showed that cyclin E expression is associated with overall recurrence but not with response to platinum-based chemotherapy (recurrence before six months). Studies that included non-chemonaive patients did not observe an association between overall survival and cyclin E [30,62,64] suggesting that the chemotherapeutic treatment may impact cyclin E expression and cell cycle regulation. In some multivariate studies including a majority of serous samples, cyclin E is an independent variable of prognosis [60,61]. Interestingly, cyclin E is less expressed in serous tissues than other histosubtypes [57,61] which again suggests that the prognostic value of cyclin E should be analyzed in individual EOC histosubtypes. Indeed, it is likely that complex mechanisms contribute to loss of cell cycle control among these different histological subtypes [30]. Nevertheless, cyclin E is a promising biomarker candidate of ovarian cancer outcome, since it has shown the most reproducible results compared to other candidates.

**Cyclin D1** expression has been linked to poor patient outcome, however some studies also reported no association with outcome in serous cohort of patients (review in [12], Supplementary file). Cyclin D1 expression is not related to disease features such as stage, grade, or histopathology [64,65]. In high stage disease (III and IV) an association between cyclin D1 protein expression and patient survival has been reported [64,66]. Studies using cohorts with mixed subtypes of EOC did not support a relationship between survival and cyclin D1 expression [57,65,66,67,247]. 

**Cyclin D3** was determined to be a prognostic marker in a reported study of 82 patients, representing different histologic subtypes, grade and stages of the disease [68]. In contrast to cyclin D1 and cyclin E, the presence of cyclin D3 was reported to be associated with better patient survival (110 months compared to 22 months in patients with no expression of cyclin D3).

**Cyclin A** has been found to be overexpressed in some ovarian tumors, however overexpression and assocation with patient survival remains controversial, mainly due to the limited number of patients and lack of well defined quality criteria in these studies. In a cohort of 31 patients, Yoons *et al.* [69] reported an association between high expression of cyclin A and poor three-year survival rates. On the other hand, Davidson *et al.* [70] reported a better overall survival of patients with high expression of cyclin A in effusion samples of high stage disease. The differential expression of cell cycle regulators is also linked to the histopathological type of the EOC (review in [71] and Supplementary file). An association of cyclin A and patient survival of patients has been observed in endometrioid tissues but not in serous tissues [72]. New studies are required to clarify the significance of cyclin A as a prognostic indicator of ovarian cancer.

#### 2.3.2. Cyclin Inhibitors: p21, p27, p57 and p16

The activity of cyclin E is inhibited by the cyclin inhibitors p21^Waf1/CIP1^, p27^Kip1^ or p57^kip2^ while the activity of cyclin D is inhibited by p16. The negative regulation of cyclin D or E leads to cell cycle arrest in G1 phase. The predictive value of p21^Waf1^ and p27^Kip1^ have been analyzed in ovarian cancer patients (reviewed by [12]). In serous tumors, positive p27^Kip1^ staining is significantly higher in early stage than that in advanced stage disease (p = 0.03, Fisher's exact test). In several studies, Kaplan-Meier curves and log-rank testing showed that absence or low p27^Kip1^ expression significantly correlates with poor survival of patients in univariate or multivariate analyses [32,59,64,73,74,75,76]. In contrast, Schmider–Ross *et al.* [76] reported, in a cohort of 165 patients, that high p27^Kip1^ expression is associated with longer disease-free progression, but that this marker did not predict responses to either taxol- or platinum-based chemotherapy. Similar results were found in a cohort of 185 patients with advanced stage disease [77]. However, an association between p27^Kip1^ and chemotherapy response was not observed in other studies including all grades, stages and histopathologies [62,78]. Interestingly, Rosen *et al.* [79] also analyzed the cytoplasmic expression of p27^Kip1^, instead of the usual nuclear expression, which represents the inactive status of p27^Kip1^. The authors reported a strong correlation with shorter disease-specific survival. 

A number of studies have reported controversial results (see review in [12] and Supplementary file) about the prognostic value of p21^Waf1^. The p21^Waf1^ protein was either reported to be associated [30,45,64,80,81,82,83] or not to patient survival or chemotherapy response [16,35,77,84,85]. Since wild-type p53 regulates the transcription of the p21 gene, the protein level of p53 may influence the level of p21 and account for the prognostic value of p21^Waf1^. A correlation between p53 and p21^Waf1^ expression was observed in most [35,64,78] but not all studies [82,83,84]. In addition, the prognostic value of p21^Waf1^ was reported to be dependent on wild type expression of p53 [78,80,84], which may explain some of the discrepancies between previous analyses. Altogether, the prognostic significance of p21^Waf1^ in ovarian cancer still remains unclear.

Few studies have reported the association of p57^kip2^ and the prognosis of ovarian cancer patients. Sui *et al.* analyzed 47 patients with malignant ovarian cancer and found a significantly lower survival rate associated with low p57^kip2^ expression [86]. However, this result was not reproduced in two other studies that included a larger number of patients [22,57]. 

Consistent with the association between cyclin D1 and poor outcome observed in some studies, the loss of p16^Ink4A^ protein expression has also been linked to a reduced five-year survival rate in a study analyzing 300 ovarian cancer patients of mixed subtypes treated with palictaxel and platinum [87] as well as in a smaller cohort of 43 patients [88]. However, in the subgroup of optimally debulked patients, p16^Ink4A^ was no longer associated with the rate of patient survival [87]. Even if no correlation was observed between the expression of p16 and FIGO stage [65,87,89], p16 loses its significance as a prognostic variable in tumors of high stage disease [30,64,88]. Conversely, p16 expression has also been linked to a worse prognosis in patients [22,90,91]. Although puzzling, Dong *et al.* observed an inverse prognostic value comparing stromal and epithelial p16 expression [91]. In the stromal cells from 159 ovarian tumor tissues from patients presenting a range of grades, stages and histopathologies, high p16 expression was associated with a better overall survival while high expression in tumor epithelial cells is linked to a shorter survival. 

### 2.4. Apoptosis

**Figure 2:**
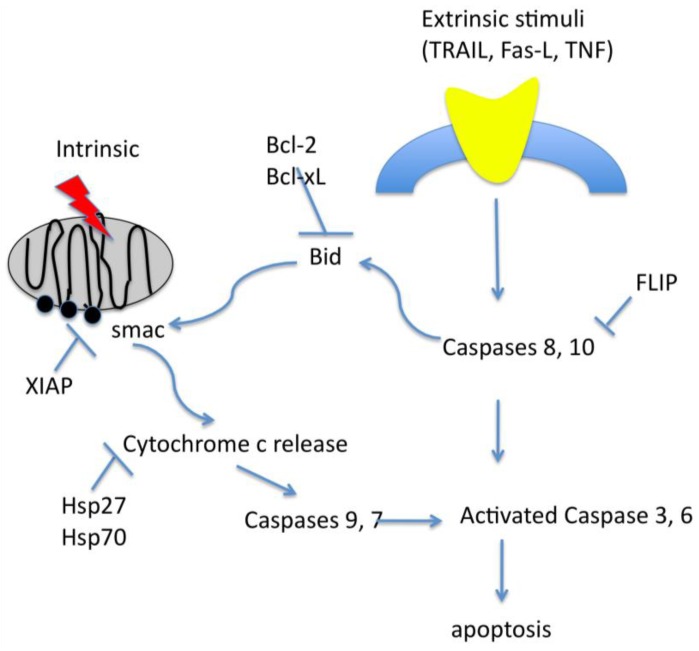
Brief schematic representation of intrinsic and extrinsic apoptotic pathways.

Apoptosis is programmed cell death chracterized by specific cellular and morphological changes. These changes are mediated by two major pathways, both leading to the release of cytochrome c and the activation of caspases, a family of cysteinyl aspartate-specific proteases. The extrinsic pathway is initiated by external stimuli, such as TRAIL, TNF (Tumor Necrosis Factor) and Fas-L, while the intrinsic pathway is initiated by intracellular signaling events such as p53 and Bcl-2 family protein activation (Figure 2). 

#### 2.4.1. Extrinsic Apoptotic Pathway

##### 2.4.1.1. TRAIL and Receptors

TRAIL, TNF Related Apoptosis Inducing Ligand, is an extracellular protein that triggers apoptosis through binding to a family of membrane receptors leading to the activation of caspase 8 and 3. Two categories of TRAIL receptors exist. TRAIL-R1/DR-4 and TRAIL-R2/DR-5 belongs to the first category and contain a death domain capable of inducing apoptosis while TRAIL-R3/DcR1, TRAIL-R4/Dcr2 and TRAIL-R5, also called decoy receptors, lack a functional death domain. Expression of different combinations of TRAIL receptors has been shown to enhance the cellular killing abilities of TRAIL in a variety of cell lines (review in [92] and Supplementary file). TRAIL is expressed in tumor and stromal cells. Only in high stage (III-IV) patients is the presence of stromal TRAIL significantly associated with survival in univariate and multivariate analysis [93]. However, even if no differential expression was reported between pre-and post- treated samples, the association between TRAIL and survival was not observed in platinum-treatment patients with either high stage or low stage disease [94]. In a restricted analysis of serous patients, Ouellet *et al.* also reported the absence of any significant association between TRAIL and disease-free survival at 18 months [58]. Since TRAIL-induced apoptosis can be regulated at the level of receptor and decoy receptor, evaluation of TRAIL and its receptors has also been done but no receptor was associated with patient prognosis [58,93]. 

TRAIL-induced apoptosis is also regulated by the cellular FLICE inhibitory protein (FLIP), which can block caspase-8 cleavage due to their structural similarities. Two forms of FLIP, named FLIPs and FLIP(L), have been identified to date, both of which contain two death domains. The level of stromal or tumoral FLIP alone does not show an association with survival rate of patients [58,93,94], but in concert with Dr4, Dr5 or p53, low levels of FLIP(L) correlates with a worse patient prognosis [58,95]. 

##### 2.4.1.2. Fas and Fas-L

The Fas (CD95) family of cell surface receptors initiates an apoptotic pathway through interaction with FasL. Upon activation, the Fas receptor trimerizes and interacts with FADD to form the death inducing signaling complex (DISC), which drives downstream events leading to the activation of caspase 8 and caspase 10. Very little information is currently available for Fas and Fas-L in ovarian cancer cells. A study of 35 ovarian carcinomas showed that Fas-L is overexpressed compared to benign or borderline tumors, and surprisingly, this overexpression was linked to a poorer five-year survival rate. However, due to the very limited number of patients studied, this observation needs to be reproduced in larger patient cohort [96]. In 75 peritoneal and pleural effusions, Dong *et al.* [97] also observed that tumor cells expressing high levels of Fas conferred a poor response to chemotherapy, including platinum treatment, in ovarian cancer patients, but was not associated with disease-free progression survival or overall survival. In contrast, platinum-based chemotherapy treatment reduces Fas expression levels [94]. The presence of Fas in tissues obtained after platinum treatment correlates with disease stage and longer disease-free and overall survival, but not in multivariate analyses [94]. 

#### 2.4.2. Intrinsic Apoptotic Pathway

##### 2.4.2.1. The Bcl-2 Family Members

Defects in apoptotic pathways can result in reduced sensitivity to chemotherapeutic drugs. Expression of the gene products involved in pro- and anti-apoptotic pathways have been implicated in patient outcome and drug resistance in ovarian cancer. This includes p53, which enhances cell death through regulation of the Bcl-2 protein family. More than 20 members of the Bcl-2 family have been identified, including proteins that suppress (Bcl-2, Bcl-xL, Mcl-l, Bcl-W, Bcl-G) and proteins that promote (Bax, Bak, Bad, Bid, Bik, Bim, Bcl-xs) apoptosis. Diverse Bcl-2 family proteins are capable of physically interacting, forming homo- or hetero-dimers, a property critical for their ability to regulate each other. 

**Bax**, a pro-apoptotic Bcl-2 member, leads to the release of cytochrome c and activation of caspase 9 and 3. In line with the *in vitro* evidence of the role of Bax, its expression has been associated with apoptosis in ovarian cancer tissues [98] and patients survival. In a cohort of 52 patients representing a range of histopathology and tumor grades, Schuyer *et al.* found that patients expressing high levels of Bax in tumors had a significantly longer median survival (57 months) than patients with low Bax expression (18 months) [85]. Similar results were obtained when the progression free survival was analysed. Interestingly, in a independent and larger cohort of 185 women with stage III disease, Bax was also associated with better survival [77] as well as in a cohort of 102 patients with early stage disease and post-treated with radiotherapy [99]. The relationship between p53 and Bax expression remains unclear. While most studies have not observed a correlation between Bax and p53 protein levels, [44,100,101,102] at least one study has reported such an association [98]. Bax was associated with survival of early stage patients in a p53 mutant subgroup but not in the p53 wild type subgroup [100]. In contrast, others observed better survival in patients with both high Bax and p53 protein accumulation levels [101]. On the other hand, Bax expression was associated with complete remission in 199 patients, but not when patients were separated into p53 mutant and p53 wild type subgroups [102]. Although the number of samples was very limited, it was noted that mucinous carcinomas showed the lowest rate of Bax staining while clear cell carcinomas had the highest rate [98,99] suggesting that Bax should be analyzed separately in each histologic subtype. 

**Bcl-2** itself has *in vitro* an anti-apoptotic activity and has been inversely correlated with the level of apoptotic index in ovarian cancer tissues [98,103,104]. Paradoxically, some studies showed an improved survival in patients with high Bcl-2 expression [98B,^104^,^105^], and improved disease-free survival with high expression of Bcl-2 [98,101], in contrast to most studies that did not report a significant association between Bcl-2 expression in ovarian cancer tissues and prognosis [20,44,80,85,96,100,102,106,107,108,109,110,111,112]. Other conflicting results indicate that patients with high Bcl-2 levels show the lowest frequency of complete response to chemotherapy, and a shorter survival, when treated with platinum-based regiments [101,103,113]. 

Bcl-2 and Bcl_XL_ are homologous proteins and share similar functions as apoptosis suppressors. However, the expression pattern of these proteins is strikingly different and Bcl-2 has been more extensively studied as a molecular marker than Bcl_XL_. The expression of Bcl_XL_ increases after chemotherapy treatment [82,114], and this variable, although not always well detailed in published reports, may affect its prognostic value. Limited studies have linked Bcl_XL_ to shorter disease-free progression but not to overall survival [77,82,110,113]. 

The other anti-apoptotic Bcl-2 familly members, such as Mcl-1, Bcl-W or Bcl-G have not been extensively investigated in ovarian cancer. No differential expression has been observed in malignant tumors compared to benign or normal tissues [115,116]. Positive staining of Mcl-1 in a cohort of 185 heterogenous patients displayed a significant association in univariate but not multivariate analyses of five-year survival rates [77], but this observation needs to be validated in independent studies. 

##### 2.4.2.2. Caspases

There are two categories of caspases: initiator or effector, depending or their role in the apoptotic signaling cascade. Caspase-3/CPP32, -6 and -7 are effectors while caspase-8, -9 and -10 are initiators. Activated caspase 3 coordinates the DNA fragmentation, which leads to cell death. Results reporting on the association of caspase 3 and the outcome of ovarian cancer patients are conflicting. In serous pleural and peritoneal effusions the level of cleaved caspase 3 and 8 correlated with improved progression free and overall survival although in multivariate analysis, only cleaved caspase 3 was an independent variable [117]. However, in serous primary solid tumors the level of cleaved caspase 8 is not associated with patient survival [58] and similarly, it appears to lose prognostic significance in samples obtained post-chemotherapy [94]. In contrast, one study has reported that caspase 3 was associated in both univariate and multivariate analyses with shorter disease-free progression and survival in 43 pre-chemotherapy and 36 post-chemotherapy patients with stage III disease [82]. 

##### 2.4.2.3. Inhibitor of Apoptosis: IAPs

Inhibitor of apoptosis proteins (IAPs) are caspase inhibitors that prevent apoptosis by specifically inhibiting caspases 3, 7, and 9. This family of proteins contains eight members: cellular IAP1 (c-IAP1), cellular IAP2 (c-IAP2), neuronal apoptosis inhibitory protein (NAIP), Survivin, X-linked IAP (XIAP), Apollon, testis- specific IAP (Ts-IAP), and Livin. Livin is not expressed in ovarian cancer cells [118]. In ovarian cancer, Survivin is the best known member of the IAPs family. It is overexpressed in high-grade tumors compared to low grade tumors [110,118]. Nuclear expression was associated with better disease-free survival and overall survival in tumor cells from samples derived from 101 effusions. However, this association is lost in samples obtained after chemotherapy [118]. In contrast, in primary solid tumor samples, the expression of Survivin was associated with poor overall survival [119]. No information is available on the predictive role of the other IAP family members with the exception of XIAP, which in one study, including 101 patients with a majority of serous EOC subtype, was found to have no correlation with survival [118]. 

### 2.5. Repair Enzymes: BRCA-1 and -2, PARP, ERCC1

The polyADP-ribose polymerase 1 (PARP-1) is a repair enzyme involved in the single strand DNA break repair pathway and cell apoptosis, while BRCA-1 and -2 are involved in double strand break DNA repair by homologous recombination. So far, only one report has investigated the level of PARP expression and its association with response to standard chemotherapy in serous ovarian cancer patients [120]. 

Excision repair cross complementation (ERCC1) is a key protein in the excision of DNA adducts caused by platinum compounds. ERCC1 is expressed in less than 15% of EOC tissues analysed and increases after platinum treatment [121]. Positive staining of ERCC1 is associated with platinum-based chemotherapy resistance [121,122], shorter disease-free survival and with a trend for shorter overall survival [121]. Interestingly ERCC1 was still significantly associated within a multivariate analysis including age, disease stage, grade and residual disease. This study validates the association between ERCC1 mRNA expression and poor outcome of ovarian cancer patients observed in several other studies [123,124]. 

The role of BRCA-1 and -2 are most often investigated in the context of hereditary ovarian cancer, but recent reports also highlight the relatively high prevalence of somatic mutations in BRCA-1 and -2 genes and the potential use of individualized therapy for these patients [125,126,127]. In additon, evidence suggests a decrease in BRCA-1 protein expression with advancing stage as seen by immunohistochemistry [128]. In the same study of 230 patients, the decreased expression of BRCA-1 (<10%) also correlated with the serous subtype, longer progression-free survival and longer overall survival [128]. However, this association was not seen in a smaller cohort of 87 Thai patients [129], therefore the potential of BRCA as a predictive marker in the general ovarian cancer population requires further investigation. 

### 2.6. Markers of Angiogenesis

#### 2.6.1. Markers of Microvascular Density

Tumors secrete extracellular mediators that initiate the growth of blood vessels, thus allowing increased tumor vascularization. This process, named angiogenesis, has been associated with an unfavorable prognosis. In addition, inhibitors of angiogenesis have shown promise as therapeutics in ovarian cancer [130,131,132]. However, the complete clinical evaluation of these new agents and effective combinations of these agents with standard treatment for a particular disease are still lacking. 

Angiogenesis assessment is often performed by immunohistochemistry with the method of Weidner *et al.* [133], where endothelial cell markers are stained to estimate microvessel density. In ovarian cancer, several reports have linked microvessel density and outcome in ovarian cancer. Although vessel density is heterogenous in tumor tissues, higher densities were observed in malignant tumors compared to borderline and in high stage tumors compared to low stage tumors [134,135]. A few additional markers, such as CD31, CD34, factor VIII and CD105, are also used to estimate angiogenesis. CD31/PECAM1 is expressed on circulating platelets and act as a major constituent of the endothelial cell junction. CD31 is the most commonly used marker in immunohistochemical analyses to assess microvessel density (also called MVD) and tumor angiogenesis in tissues. It is considered as a more specific marker of endothelial cells than factor VIII. Evaluation of MVD, as determined by CD31, has also led to largely contradictory results concerning the outcome of ovarian patients. In several studies, increased MVD compromised the overall survival in a cohort of late stage patients when high MVD was compared to low MVD [136,137,138,139]. Other studies, based on more limited cohorts of late stage disease, observed a better overall survival in patients with high MVD [140,141]. While no correlation between overall survival and MVD was found in 77 patients with stage I disease [142], the same authors showed that patients with high MVD had a shorter disease-free interval. In contrast to this result, when MVD was assessed with CD34, a better disease-free interval was observed in stage I patients with high MVD [143]. Evaluation of MVD, as determined by CD34, has also led to contradictory results [138,141,144,145,146,147,148,149,150,151,152,153]. Heterogeneity of ovarian tumors and measurement techniques may interfere with the assessment of the prognostic value of MVD in these tissues. In addition, the lack of methodology standardization, small and mixed cohort selection, and variation in treatment and outcome data make these studies very difficult to compare.

#### 2.6.2. Markers of Proteins Involved in Angiogenesis

**VEGF** plays a major role in proliferation and migration of endothelial cells, thereby nourishing and favoring tumor growth. In tumor tissues, VEGF is expressed by epithelial tumor cells, stromal cells and macrophages [154]. It is significantly overexpressed in ovarian carcinomas as compared to benign or borderline tumors and this expression is inconsistently associated with disease stage and tumor grade [154,155,156]. In some studies, the survival rate of patients with high VEGF expression was worse than for patients with low or no VEGF expression [151,154,155,156,157,158] even in stage I patients [142]. This association remained significant in some multivariate analyses [154] but not in every study [155,156] and not in stage I patients [142]. In contrast, no association between VEGF expression in tissues and progression-free survival, response to chemotherapy or overall survival, was seen in cohorts composed of only cancer patients with advanced disease [143,159,160]. A larger study analyzing VEGF expression in 320 patients showed that patients with high VEGF expression in tumor tissues had a median survival of 24 months while patients with low VEGF expression had a median survival of 13 months [161]. Similar results were obtained by O’Toole in a cohort of 79 patients [162]. This association was also significant in the multivariate model including age, residual disease and stage. With respect to serum VEGF, a number of studies also reported an association with poor prognosis. These studies have been used in a meta-analysis pooling 314 patients and confirmed the relationship between high serum VEGF and poor overall survival [163]. Another study, which determined the level of VEGF in 39 ascites fluids, observed that high levels confer a shorter disease-free survival and overall survival than low levels [157]. In contrast, two studies were not able to reproduce the association between VEGF and survival of ovarian cancer patients. In a cohort of 112 patients VEGF was associated with better disease-free progression but not overall survival [152]. Similarly, western blot analysis of 67 high stage ovarian cancer tumors did not show any association between the level of VEGF, or its receptor, and outcome (disease-free progression or survival) of patients [159]. 

**Figure 3:**
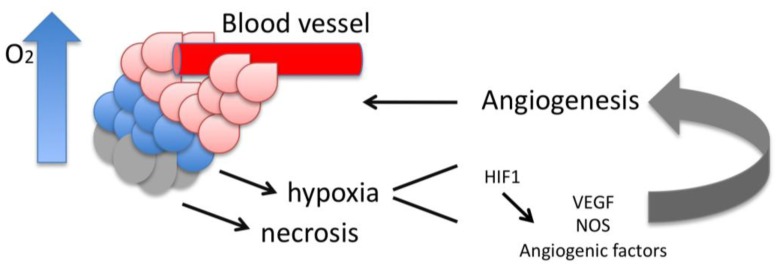
Role of hypoxia in tumor angiogenesis.

**HIF**, hypoxia-inducible factor, is a transcription factor involved in several cellular processes and is a key regulator of tissue hypoxia. It increases O_2_ availability and favors tumor growth and neoangiogenesis. It likely acts by increasing the expression of genes, such as VEGF and Nitric oxide synthetase, that regulate hypotoxic stress and angiogenesis (see Figure 3). HIF is overexpressed in ovarian cancer tissue as compared to benign tissue [149,164,165] and closly correlates with microvascular density of cancer tissues [149]. Among the different histological types of ovarian carcinoma, HIF is more highly expressed in clear cell carcinoma [165]. With the exception of one initial study [149], immunohistochemistry reveals that higher nuclear HIF-1 presence is associated with a poorer survival (45 compared to 70 months) in patient cohorts representing a range of histopathologies and stages, including advanced stage of serous disease [152,164,166]. This association remained significant in multivariate analysis [166]. In contrast, using a Western blot assay, it has been reported that in 52 patients with sub-optimally debulked stage III/IV EOCs, which were further treated with a combination of Taxol and Carboplatin, HIF-1α expression correlated with significantly better survival [167] although this was not the case for the optimally debulked patients. 

**Cyclooxygenase-2 (COX-2)**, also known as prostaglandin-endoperoxide synthase-2, regulates prostaglandin synthesis and is involved in several cellular and biological processes, such as inflammation, apoptosis, proliferation, neoplastic effects, regulation of metalloproteinase and angiogenesis. Cox-2 is not expressed in normal ovarian epithelia [168,169]. In ovarian cancer tissues, Cox-2 expression positively correlates with the microvascular density of tumors [150,151,170,171] confirming its role in angiogenesis of ovarian tumors. Most studies report an association between a poor prognosis in ovarian cancer patients and high Cox-2 expression [168,169,170,171,172,173,174,175,176]. Ali-fehmi *et al.* [170], who assessed 117 advanced stage serous ovarian carcinoma specimens from patients who did not received chemotherapy prior to surgery, reported that high Cox-2 expression is predictive of a short survival. This association was confirmed in a large study of 442 serous patients for which a five-year follow up was available [168]. Ferrendina *et al.* [177] observed such an association only in patients who underwent explorative laparatomy but not in patients that underwent primary debulking. In strong contradiction with all these results is another study analyzing 160 patients with long term follow-up (10 years), which reported a better survival rate in patients with high Cox-2 expression, and if correct suggests that Cox-2 may have different biological effects during the progression of the disease [178]. On the other hand, the expression and predictive value of Cox-2 differs between mucinous and non-mucinous tissues [169], which could be a variable influencing its predictive value. In addition, Cox-2 expression is not significant in all multivariate analyses reported [169,173]. 

Thrombospondin 1 (THBS-1) is a homotrimeric secreted glycoprotein with an anti-angiogenic effect. It acts by inhibiting neo-vascularization. In all studies except one [159], which included a mixed group of histopatholic types, THBS-1 was not related to survival [142,179,180], either in low stage patients or advance stage patients. 

**Metalloproteinase** (**MMP**) regroups a large family of zinc- and calcium-dependent enzymes able to degrade the extracellular matrix and thereby facilitate invasion and metastatic spread [181]. A few studies have associated MMP expression with a poor prognosis in ovarian cancer (reviewed in [12,182,183] and Supplementary file). In particular, MMP-9 was investigated in a large cohort of 292 EOC patients. High stromal expression of MMP-9 was associated with poor prognosis, although this association was not seen when comparing expression within tumor cells [184]. On the other hand, the tumoral expression of MMP-7 is an independent prognostic factor, predicting better survival and disease-free survial in ovarian cancer [185].

### 2.7. Immunological Factors

A large number of studies have reported an association between outcome and chemosensitivity of ovarian cancer patients and the presence of tumor-infiltrating immune cells or immunological factors. For example, the presence of T cells is associated with a good prognosis or chemosensitivity in ovarian cancer patients [186,187,188,189,190,191,192,193,194] while the presence of inhibitory T-Regs cells is often associated with a shorter survival [195,196,197,198,199], although not reproduced by all [200,201]. The survival rate of ovarian cancer patients has also been correlated with the presence of cytokines, co-stimulatory or inhibitory molecules expressed by immune cells or tumor cells [188,193,198,200,202,203,204]. For a detailed review see [193].

### 2.8. Tyrosine Kinase Receptors (TKR)

#### 2.8.1. Epidermal Growth Factor Receptors: ErbB

One of the most studied tyrosine kinase receptor families is the epidermal growth factor receptor (EGFR). This family of proteins has been extensively studied in breast, lung, colon and prostate cancer and its members include EGFR/ErbB1, Her-2/ErbB2, Her-3/ErbB3 and Her-4/ErbB4. EGFR is more often related to poor survival of cancer patients, albeit unconsistently, while the ErbB2 prognostic significance remains uncertain. Similar results were obtained in ovarian cancer tissues (reviewed in [11,12] and Supplementary file). A recent meta-analysis of the literature using different studies including diverse patient cohorts concluded that EGFR and Her-2 have a limited influence on patient outcome, although their broad conclusion highlights the fact that studies need to be restricted to specific subtypes of ovarian cancer [11]. There are limited reports on the prognostic significance of ErbB3 and ErbB4 in ovarian cancer. ErbB3 expression was initially associated with a poor outcome in 28 endometrioid ovarian cancer patients [205] and more recently others reported a similar result in a cohort of 116 patients representing the range of histpathologies, stage and grade of the disease [206]. Although the study of Her-2 has not proven useful in most ovarian carcinoma subtypes, Her-2 overexpression and amplification can be seen in 20% of mucinous carcinomas and these cancers may respond to therapy targeting this TKR [207]. 

#### 2.8.2. Ephrin B receptors and other TKR

More recently, the role of Ephrin (EPH) receptors has been investigated in ovarian cancer. The EPH receptors comprise a large family of TKR divided into the EphA and EphB subfamily, based on their sequence and structure. The activities of Eph receptors includes diverse cellular functions such as immune regulation, embryonic cell movement and tumorigenesis. In tumorigenesis, Eph receptors are suspected to promote angiogenesis, proliferation and invasion [208]. Particularly, EphB4 expression is confined to epithelial cells and has been reported to be expressed in EOC cells but not in normal ovarian epithelia [209]. High expression of Ephb4 is associated with poor reponse to chemotherapy in 72 patients with advanced stage disease [210], as well as poor survival rates in two independent cohorts including a range of EOC diseases [209,211]. Interestingly, EphB4 remains an independent variable in multivariate analysis [209]. While promising, these results need further investigation on a larger cohort of patients. 

Similar to EGFR receptors, the high expression of hepatocyte growth factor receptor (Hgf/Met) and its ligand, c-Met, has been associated with a shorter overall survival of ovarian cancer patients [212,213]. 

#### 2.8.3. The Signaling Pathway of Tyrosine Kinase Receptors: Akt and NF-κB

Signaling pathways involved in apoptosis and cellular survival, such as Erk, Akt, PI3K, Akt, NF-κB have also been investigated for their potential association with the outcome of ovarian cancer patients [31,214,215,216,217,218,219]. Contradictory results have been reported. The PI3K/Akt pathway is a major signaling pathway activated by tyrosine kinase receptors. Activated Akt promotes downstream signaling involved in cell growth, proliferation and cell survival. In a recent study involving 63 patients, the presence of active/phosphorylated Akt was correlated with the presence of nuclear p65 and was associated with a lower rate of survival at five years [214]. The presence of active Akt was more significantly associated with reduced survival of patients as compared with the presence of nuclear p65 (p = 0.009 and p = 0.04, respectively). The association between nuclear NF-κB and poor survival was also observed in effusions from 166 patients [117]. However, the association between Akt and survival of ovarian cancer patients was not seen in previous studies [31,216,218]. Since these studies included either a limited number of patients and/or a range of pathologies, such studies need to be reproduced. Neither Akt nor NF-κB were significant variables in multivariate analysis [117,214].

### 2.9. E-cadherin/beta-Catenin

**Cadherins** are type-1 transmembrane glycoproteins involved in cell polarity and cellular adhesion ensuring cell adherence to tissues. Epithelial-cadherin (E-cadherin) is expressed by epithelial cells. Loss of E-cadherin results in increased cellular motility, the epithelial-mesenchymal transition and cancer progression. In EOC patients [220] as well as in serous cohorts of patients, E-cadherin down-regulation is associated with poorer survival [221,222]. However, Voutilainen *et al.* [223] did not observe a significant association of E-cadherin with overall survival in a mixed range of histopathologies but they noticed a significantly better reponse to chemotherapy treatment. Interestingly, E-cadherin expression is less pronounced in clear cell and mucinous carcinomas [223], which may influence results towards a lack of prognostic significance in mixed patient cohorts. 

The proper function of E-cadherin is regulated by its binding to catenin proteins. Binding of E-cadherin to catenins is essential for the maintenance of actin cytoskeleton and stabilizing cell-cell junctions. It is suspected that impaired function or expression of catenins may also be involved in ovarian cancer progression. Beta-catenin has been more studied that α- or γ-catenins. Furthermore, β-catenin is the target of Wnt signaling pathway. Wnt signaling results in phosphorylation of cytosolic β-catenin by Gsk-3β which leads to nuclear accumulation and trancriptional activation of target genes such as Cox-2 and cyclin D. The nuclear presence of β-catenin is significantly different among the histopatholgies of ovarian cancer [17,224] and also correlated to better survival of patients [220,224]. 

## 4. Discussion

For several years, efforts to identify reliable prognostic factors have focused on molecular markers. A large number of these markers have been investigated to date, usually by immunohistochemistry. Given the large number of potential candidate markers published (see Supplementary file for selected examples), it is remarkable that none have been approved for clinical use. Clearly, an overview of the literature shows that the determination of a common marker, or set of ovarian carcinoma markers identified in independent studies, has not been accomplished [225]. The focus on prognostic biomarkers may have been misplaced as other in very specific circumstances. Ironically, many markers published as having potential prognostic utility that are now questionable due to an association with subtypes, have proven to be useful as diagnostics to help subtype problematic cases (WT-1 in clear cell carcinoma and serous-mixed cancer) [21]. With recent clinical trial data showing potential utility for neoadjuvant therapy [8], a need for biomarkers to facilitate accurate subtyping from minimal tissue samples, *i.e.*, FNA’s or needle core biopsies, has emerged and many of the markers described above could prove to be useful for that purpose. 

Lack of reproducibility is a feature of most of these biomarker studies; this can be explained at least in part by technical and biological factors [225,226]. Two immediate explanations are apparent. Firstly, access to appropriate patients can be limited; most studies have analyzed only a small number of samples. Due to the inter-individual biological variability and heterogeneity of ovarian tumor tissues, results obtained on small sets of patients (n < 100) are often not reproducible on larger sets of patients [227]. A second explanation is that most studies include heterogenous material with different histologic subtypes, different patient treatments, as well as different grades and stages of the disease. Since ovarian carcinoma subtypes are immunophenotypically distinct entities and have different clinical features such as stage at presentation and outcome biomarkers associated with a subtype, such as biomarkers expressed in serous epithelium, will have a predictable association in studies on mixed cohorts of ovarian carcinomas [16]. In addition, when biomarker data from cohorts with different proportions of subtypes are compared, one can reasonably expect that the association of biomarkers with outcome or stage at presentation can be expected to appear irreproducible [16]. 

Study quality is also a strong parameter influencing the inter-reproducibility of biomarkers analyzed. Studies should include inclusion and exclusion criteria, detailed tumor characteristics, selection of patients, follow-up time, additional treatments and specificity of the assay used. A particular technical challenge is the reproducibility associated with probing protein expression using specific antibodies raised against different epitopes. Some antibodies may recognize epitopes that are located in post-translationally modified regions of the protein and thus these epitopes are not expressed or detected in different conditions. In addition, most of the older studies did not verify the specificity of antibodies, thus raising the possibility that detection was non-specific. Such studies reported results that were sometimes difficult to reproduce with a different antibody, again suggesting a lack of specificity in the chosen antibody. Another important factor is the antibody titer. The use of the same antibody at different dilutions can give potentially very different results. A low concentration of antibody will quantitatively detect high expression of a marker by missing any low expression. Inversely, high antibody concentrations will quantitavely detected low expression but these same levels detect high expression but the result is not quantitative. The well described example is the anti-Her-2 antibody [228]. Only low titers of anti-Her-2 will be able to identify patients with aggressive disease (high overexpression of Her-2). 

The ability to construct tissue microarrays (TMA) has signaled a new area of high-throughput analysis to aid in the validation of tissue based biomarkers. A key benefit is the ability to analyse hundreds of patients at a time, reducing costs, time and increasing the reproducibility of results by staining the whole cohort simultaneously. However, these advantages come with a few pitfalls. Since the tissue analyzed by microarray are derived from small cores, the heterogeneity within a given tumor may influence the interpretation of results. Fortunately, in large cohort studies this sampling factor is reduced by the size of the sample set. In addition, since a TMA often contains hundreds of specimens collected over decades, the time between tumor collection and TMA construction may influence the antigenicity of certain biomarkers. Furthermore, during TMA construction, tissue oxidation may occur and induce a loss of antigenicity for some antibodies [229]. 

In addition to the aforementioned technical and study design problems associated with many immunohistochemical studies of ovarian carcinomas, a lack of transparency with and accountability for primary data from such studies may have contributed to the general lack of reproducibility. Fortunately it is now almost trivial to provide image archives from TMA and other studies to enable other researchers to calibrate and compare scoring systems. Tissue specimens can be analyzed with different systems ranging from pathologist image analyses to fully automated quantitative systems. Performing cross-platform comparisons should improve the reproducibility and validity of markers [230]. Finally, the lack of good quality control and guidelines [230] is often missing. Recent published guidelines for high standard biomarker discovery [231,232,233,234] will no doubt promote high quality studies and contribute to the greater reproducibility needed to validate clinically significant biomarkers.

Although the lack of currently usable tissue-based prognostic or predictive biomarkers for ovarian cancer may give little sense of optimism, this should change over the next few years. Firstly there is a recognition that ovarian carcinoma subtypes are distinct diseases and should be treated as such in research and ultimately in the clinic. Major discovery-based biomarkers studies are now typically subtype-specifc. For instance, The Cancer Genome Atlas Project (TCGA) has performed in-depth genomic analysis of high grade serous carcinomas and has recast our understanding of that disease. In addition to improved study methodology based on a subtype-specific focus, today’s biomarker discovery research can include the decoding of ovarian cancer genomes at base-pair resolution. This approach, empowered by next generation sequencing technologies has already been used to identify a pathogenomic mutation in granulosa cell tumors and is now being applied to ovarian carcinomas [235,236]. 

There is a great need for diagnostic biomarkers for subtyping carcinomas, and predictive biomakers to identify women with high grade serous carcinomas who will not benefity from standard therapy. For the other subtypes, the development of new treatment approaches will demand the co-development of predictive biomarkers. These needs will only be met in a timely fashion if large clinically annoted cohorts are developed as communal resources for biomaker validation and if tissue banking and the collection of tumour blocks remeains a key priority in clinical trials.

While the present literature is replete with biomarkers, their true promise in changing clinical practice has not yet been realized. The most promising markers, as well as new candidates, will no doubt benefitfrom additional well-designed studies that would support their effectiveness and their usefulness in the clinical setting.

Recommendations for future studies:

- Consider EOC subtypes as different diseases for the purposes of biomarker studies.
- Clearly define and report the study cohort: inclusion and exclusion criteria for selecting patients, complete histopathology, stage and grade. Knowledge of treatment regimens is highly recommended. Recommendations addressing these points are provided by the NCI [231].
- Consider pitfalls of technology used for evaluation biomarkers: for tissue microarrays see [229].
- Apply high standard for study design, including a minimum number of patients, focusing on high statistical standard, see [232,233,234].
- Validation from independent investigators is an important criterion for determining the robustness of any given biomarker.

## Supplementary Files

Supplementary File 1 PDF-Document (PDF, 645 KB)
