# Supplementary material for: Predictive and Prognostic Protein Biomarkers in Epithelial Ovarian Cancer: Recommendation for Future Studies
Source: Cancers (Basel). 2010 May 26;2(2):913–54. doi: 10.3390/cancers2020913 (PMC3835111; doi:10.3390/cancers2020913)
Supplement: Supplementary File 1 — PDF-Document (PDF, 645 KB) [file cancers-02-00913-s001.pdf]

Cancers **2010**, *2*, 1-15 supplementary file; doi:10.3390/cancers2020913

OPEN ACCESS

cancers

ISSN 2072-6694
www.mdpi.com/journal/cancers

Review

Predictive and Prognostic Protein Biomarkers in Epithelial Ovarian Cancer: Recommendation for Future Studies

Cécile Le Page [1], David G. Huntsman [2,3], Diane M. Provencher [1,4] and Anne-Marie Mes-Masson [1,5,*]

[1] Centre de recherche du Centre hospitalier de l'Université de Montréal (CR/CHUM), Institut du cancer de Montréal, 1560 Sherbrooke Est, Montreal, H2L4M1, QC, Canada; E-Mails: cecilelepage@yahoo.ca (C.L.P.); diane.provencher.chum@ssss.gouv.qc.ca (D.M.P.)
[2] Department of Pathology and Genetic Pathology Evaluation Centre of the Prostate Research Center, Department of Pathology and Laboratory Medicine, University of British Columbia, Vancouver General Hospital, Vancouver, Canada; E-Mail: dhuntsma@bccancer.bc.ca (D.G.H.)
[3] Translational and Applied Genomics, BC Cancer Agency, Room 3427, 600 West 10th Avenue, Vancouver, V5Z 4E6, BC, Canada
[4] Département d'Obstétrique et Gynécologie, Clinique de Gynécologie Oncologie, Université de Montréal, 1560 Sherbrooke Est, Montreal, H2L4M1, QC, Canada; E-Mail: diane.provencher.chum@ssss.gouv.qc.ca
[5] Département de Medicine, Université de Montréal, 1560 Sherbrooke Est, Montreal, H2L4M1, QC, Canada

* Author to whom correspondence should be addressed; E-Mail: anne-marie.mes-masson@umontreal.ca; Tel.: +1-514-890-8000 ext 25496; Fax: +1-514-412-7703.

Received: 8 March 2010; in revised form: 19 April 2010 / Accepted: 13 May 2010 / Published: 26 May 2010

Table 1. Summary of potential predictive and prognostic protein biomarkers identified in ovarian cancer tissues

Marker	EOC patients	Histotype	CN	Treatment post-surgery	Stade	Grade	Antibody source	Study type	Follow-up (median)	Marker prediction CR	DFS	OS	ref
Akt-p	10	mixed	no	platinum-based	all	all	Cell Signaling	retrospective	-			good	[217]
Akt-p	63	mixed	yes	-	all	all	monoclonal (Cell Signaling)	retrospective	32 months			poor	[214]
Akt-p	71	mixed	-	-	all	all	587F11 (Cell Signaling)	retrospective	-			no	[31]
Akt-p	75	mixed	-	-	all	all	polyclonal (Cell Signaling)	prospective	31 months			no	[216]
Akt-p	106	mixed	-	platinum-based	all	all	monoclonal (Cell Signaling)	retrospective	72 months			trend	[219]
Akt-p	228	mixed	yes	platinum-based	all	all	-	prospective	-		no	no	[218]
Bax	44	mixed	-	-	all	all	polyclonal (Pharmingen)	retrospective	-		no	no	[110]
Bax	45	mixed	-	platinum-based	all	all	polyclonal (Pharmingen)	retrospective	21 months	good	good	no	[237]
Bax	50	mixed	-	-	all	all	polyclonal (Pharmingen)	retrospective	-		no	no	[111]
Bax	52	mixed	no	mixed	all	all	4F11 (Beckman Coulter)	retrospective	78 months		good	good	[85]
Bax	60	serous	yes	palitaxel/ carboplatin	all	all	4F11 (Beckman Coulter)	retrospective	42 months			no	[238]
Bax	93	mixed	yes	cisplatin/epirubicin/cyclophosphamide	all	all	B-9 (Tebu)	retrospective	69 months		no	no	[43]
Bax	106	mixed	-	radiation	I-II	all	polyclonal (Dako)	retrospective	87 months			good	[99]
Bax	109	mixed	-	cisplatin/cyclophos-phamide	I-II	all	polyclonal (Dako)	retrospective	48 months			good *	[100]
Bax	116	mixed	-	platinum-based	all	all	polyclonal (Dako)	retrospective	39 months			no	[108]
Bax	117	mixed	yes	cisplatin/epirubicin/cyclophosphamide	III-IV	all	B-9 (Tebu)	retrospective	68 months		no	no	[44]
Bax	140	mixed	-	palitaxel/cisplatin	all	all	2D2 (Zymed)	prospective	31 months (mean)			no	[98]
Bax	185	mixed	yes	cisplatin/epirubicin	III	all	polyclonal	retrospective	85 months	no		good	[77]
Bax	199	mixed	yes	taxane/platinum	all	all	B-9 (Santa-Cruz)	retrospective	29.4 months	poor	no		[102]
Bax	229	mixed	yes	platinum-based	all	all	B-9 (Santa-Cruz)	retrospective	24.7 months	poor			[101]
Bcl-2	35	mixed	no	mixed	all	all	clone 124	retrospective	32 months			no	[96]
Bcl-2	43	mixed	no	-	III-IV	all	2H12 (Zymed)	prospective	24 months		poor	poor	[113]
Bcl-2	44	mixed	-	-	all	all	clone 124	retrospective	-		no	no	[110]
Bcl-2	50	mixed	yes	cisplatin/ doxorubicin	all	all	clone 124	prospective	39 months			good	[105]
Bcl-2	50	mixed	-	-	all	all	clone 124	-	-		no	no	[111]
Bcl-2	51	endometrioid	-	cyclophosphamide/doxorubicin/ cisplatin	III-IV	all	clone 124	retrospective	minimum 1 month			poor	[103]

Table 1. *Cont.*

Bcl-2	60	serous	yes	palitaxel/ carboplatin	all	all	clone 124	retrospective	42 months			no	[238]
Bcl-2	66	mixed	-	cyclophosphamide/doxorubicin/ cisplatin	all	all	clone 124	retrospective	minimum 1 month	poor			[103]
Bcl-2	70	mixed	-	platinum-based	95% III-IV	all	clone 124	retrospective	84 months	no		no	[109]
Bcl-2	88	mixed	no	platinum	all	all	clone 124	retrospective	78 months		no	no	[85]
Bcl-2	90	mixed	-	mixed	all	all	clone 124	retrospective	-		no	no	[112]
Bcl-2	94	mixed	yes	platinum-based	all	all	clone 124	retrospective	24 months	poor *			[101]
Bcl-2	95	mixed	-	paclitaxel/ carboplatin	all	all	clone 124	prospective	66 months (median)	no	no	no	[106]
Bcl-2	100	mixed		Cyclophosphamide/platinum or paclitaxel/platinum	III-IV	na	na	prospective	55 months (median)	good	no	no	[239]
Bcl-2	102	mixed	-	cisplatin/cyclophosphamide	I-II	all	clone 124	retrospective	48 months		no	no	[100]
Bcl-2	103	mixed	-	cisplatin/carboplatin-based	all	all	clone 124	retrospective	60 months			no	[80]
Bcl-2	105	mixed	-	radiation	I-II	all	polyclonal	retrospective	87 months		no	no	[99]
Bcl-2	116	mixed	-	platinum-based	all	all	clone 124	retrospective	39 months			no	[108]
Bcl-2	117	mixed	yes	cisplatin/epirubicin/cyclophosphamide	III-IV	all	clone 124	retrospective	68 months		no	no	[44]
Bcl-2	119	serous	-	platinum-based	all	all	clone124	retrospective	46 months			no	[20]
Bcl-2	140	mixed	-	palitacxel/cisplatin	all	all	clone 100 (Biogenex)	retrospective	31 months		good	good	[98]
Bcl-2	185	mixed	yes	cisplatin/epirubicin	III	all	M0887	retrospective	77 months	no		good	[104]
Bcl-2	199	mixed	yes	taxane-platinum	all	all	clone 124	retrospective	29.4 months	no			[102]
Bcl-XL	28	mixed	yes	platinum-based	all	all	polyclonal DAKO	retrospective	-		poor	no	[114]
Bcl-XL	43	mixed	no	cisplatin/paclitaxel/cyclophosphamide	III-IV	all	2H12 (Zymed)	prospective	24 months		no	no	[113]
Bcl-XL	43	mixed	yes	cisplatin-based	III	all	polyclonal (Santa-Cruz)	retrospective	-		poor	trend (p=0.06)	[82]
Bcl-XL	49	mixed	-	-	all	all	polyclonal (Pharmingen)	retrospective	-		no	no	[110]
Bcl-XL	50	mixed	-	-	all	all	Dako	retrospective	-		no	no	[111]
Bcl-XL	50	mixed	-	-	all	all	Dako	retrospective	-		no	no	[111]
Bcl-XL	185	mixed	-	cisplatin/epirubicin	III	all	18-0217 (Zymed)	retrospective	85 months		no	no	[77]

Table 1. *Cont.*

□ -catenin	56	serous	yes	mixed	all	all	C19220 (Transduction laboratories)		-			good	[240]
□ -catenin	75	mixed	-	cisplatin/cyclophos-phamide	all	all	C19220	retrospective	70 months			no	[241]
□ -catenin	104	mixed	yes	mixed	all	all	C19220		-			good	[240]
□ -catenin nuclear	253	mixed	-	-	all	all	C19220		-			good	[224]
BRCA-1	87	na	na	na	na	na	na	na	43 months		no	no	[129]
BRCA-1	230	mixed	-	-	all	all	Ab-1 (Oncotech)	-	-		poor	poor	[128]
caspase 3	43	86% serous	yes	cisplatin-based	III	all	Ab-4	retrospective	-		poor	poor	[82]
caspase 3	52	serous	no	mixed	III-IV	all	polyclonal (Cell signaling)	retrospective	27 months		good	good	[117]
caspase 8	52	serous	no	mixed	III-IV	all	polyclonal (Cell signaling)	retrospective	27 months		good	good	[117]
caspase 8	179	serous	yes	platinum-based	all	all	polyclonal (Santa-Cruz)	retrospective	31 months			no	[58]
caspase 8	141	mixed	no	platinum-based	all	all	1C12 (Cell Signaling)	prospective	29 months		no	no	[94]
CD105	58	mixed	-	-	all	all	clone 105CO2 (Neomarker)	retrospective	34 months			poor	[139]
CD105	106	mixed	yes	platinum and/or taxane	III_IV	all	SN6H (Dako)	retrospective	158 months		poor	no	[242]
CD31	20	serous	-	-	all	all	JC/70A (Dako)	retrospective	21 months		no	no	[134]
CD31	28	mixed	-	mixed	III	all	Biogenix	retrospective	106 months			poor	[138]
CD31	33	mixed	yes	platinum-based	Ic	all	Dako	retrospective	73 months		poor	no	[142]
CD31	49	mixed	na	platinum-based	III-IV	na	na	retrospective	na	good	good	good	[160]
CD31	58	mixed	-	-	all	all	JC/70A (Dako)	retrospective	34 months			poor	[139]
CD31	60	mixed	yes	na	III-IV		na	na	na	poor		poor	[136]
CD31	77	mixed	-	-	all	all	JC/70A (Dako)	retrospective	-			poor	[243]
CD31	79	mixed	-	platinum-based	all	all	JC/70A (Dako)	retrospective	-		poor	poor	[162]
CD31	85	mixed	-	mixed	III-IV	all	JC/70A (Dako)	retrospective	7.5 years			poor	[137]
CD31	106	mixed	yes	platinumand/or taxane	all	all	JC/70A (Dako)	retrospective	158 months		no	no	[242]
CD31	149	mixed	-	-	III-IV	all	JC/70A (Dako)	retrospective	-	no			[135]
CD34	22	clear cells	-	cisplatin/ adriamycin and cyclopho-sphamide	I-II	all	monoclonal (Novocastra)	-	-		good		[143]
CD34	33	mixed	-	platinum-based	III	G2-3	M7165 (Dako)	retrospective	37 months			no	[180]
CD34	38	mixed	na	na	all	all	na	na	na			poor	[147]
CD34	43	mixed	-	mixed	high	all	Amac	retrospective			poor	no-trend	[146]
CD34	44	mixed	-	-	III-IV	all	monoclonal (Vantana)	-	-			good	[141]

Table 1. *Cont.*

CD34	44	mixed	-	mixed	all	-	QBEnd/10 (Biogenex)	retrospective	minium 6yrs			poor	[138]
CD34	45	mixed	-	cisplatin/adriamycin and cyclopho-sphamide	I-II	all	Monoclonal (Novocastra)		-		good		[143]
CD34	60	mixed	-	cisplatin, adriamycin and cyclopho-sphamide	III-IV	all	monoclonal (Novocastra)		-		no		[143]
CD34	60	serous	no	cisplatin-based	III	G3	QBEnd/10 (Biogenex)		-		poor	poor	[153]
CD34	63	mixed		mixed	all	all	QBEnd/10 (Biogenex)	retrospective	81 months			poor	[148]
CD34	83	serous	-	platinum-based	III	G3	QBEnd/10 (Biogenex)	retrospective	45 months		poor	poor	[151]
CD34	101	mixed	-	platinum-based	III-IV	all	monoclonal	retrospective	65 months		good	good	[244]
CD34	102	mixed	-	platinum/taxol	all	all	QBEnd/10 (Biogenex)	retrospective	28 months			no	[149]
CD34	112	serous	-	mixed	all	all	QBEnd/10 (Biogenex)	retrospective	-			good	[152]
CD34	132	serous	-	mixed	all	all	clone My10 (Becton)	retrospective	-		poor	poor	[144]
CD34	116	mixed	-	mixed	III-IV	all	clone My10 (Becton)	retrospective	23 months		no	poor	[145]
CD34	174	mixed	-	mixed	all	all	clone My10 (Becton)	retrospective	23 months		no	no	[145]
Cox-2	30	mixed	no	cisplatin-based	III	all	monoclonal (Cayman Chemical)	retrospective	-		no	no	[175]
Cox-2	43	mixed	yes	cisplatin-based	III	all	monoclonal (Cayman Chemical)	retrospective	-		poor	no	[175]
Cox-2	64	mixed (mucinous, non-mucinous)	-	cisplatin-based without palictaxel	all	all	Transduction Laboratory	retrospective	56 months			poor	[169]
Cox-2	78	serous	-	platinum-based	III	G3	polyclonal (Santa-Cruz)	retrospective	32.5 months		poor	poor	[245]
Cox-2	79	-	-	platinum-based	-	-	-	-	-	no		no	[246]
Cox-2	86	all	-	-	all	all	monoclonal (Cayman Chemical)	retrospective	32 months (mean)			poor	[176]
Cox-2	87	mixed	-	cisplatin-based	III_IV	all	polyclonal (Cayman)	retrospective	25 months			poor	[177]
Cox-2	96	serous	yes	-	III-IV	G2-3	H62 (Santa-Cruz)	retrospective	-			poor	[171]
Cox-2	100	mixed	yes	platinum-based	all	all	CX-294 (Dako)	retrospective	67 months			poor (5yr)	[173]
Cox-2	117	serous	yes	-	III-IV	G3	H62 (Santa-Cruz)	retrospective	1.7 years			poor	[150]

Table 1. *Cont.*

Cox-2	160	mixed	-	carboplatin-based	all	all	CX-294 (Dako)	retrospective	at least 10 years			good	[178]
Cox-2	442	serous	-	mixed	all	all	monoclonal 160112	retrospective	5 years			poor	[168]
cyclin A	31	mixed	-	carboplatin/ paclitaxel	all	all	monoclonal (Dako)	prospective	36 months			poor (3yr)	[69]
cyclin A	52	mixed	yes	mixed	all	all	NCL-cyclinA (Novocastra)	retrospective	minimum 10 years			no	[41]
cyclin A	55 effusions	mixed	no	-	III-IV	all	-	retrospective	22 months			good	[70]
cyclin D1	32	mixed	no	platinum-based	all	all	Monoclonal (Santa-Cruz)	retrospective	-		poor	no	[67]
cyclin D1	50	serous	-	platinum-based without paclitaxel	all	all	DCS-6 (Ventana Medical Systems)	retrospective	-			no	[247]
cyclin D1	53	mixed	yes	platinum-based	II-III	all	DCS-6 (Ventana Medical Systems)	retrospective	-			no	[57]
cyclin D1	59	mixed	-	cyclophosphamide/adriamycin/platinum	all	all	monoclonal (Medical and Biological Laboratories)	-	22 months			no	[65]
cyclin D1	70	mixed	na	na	all and III-IV		na	na	na			poor	[66]
cyclin D1	134	serous	-	-	85% III-IV	all	DCS-6 (Ventana Medical Systems)	retrospective	29.8 months		no	poor	[64]
cyclin D3	92	mixed	yes	mixed	all	all	DCS-22 (Vector Laboratories)	retrospective	4.51 months			good	[68]
cyclin E	75	mixed	no	platinum-based (100%) with taxane (83%)	III-IV	high	HE12 (Santa-Cruz)	retrospective	28 months (minimum 6 months)	no	p	no trend	[62]
cyclin E	77	mixed	-	65% platinum	all	high	13A3 (Novocastra)	retrospective	16 months			poor	[30]
cyclin E1	47	mixed	yes	platinum-based	all	all	Polyclonal (Santa-cruz)	retrospective	24 months			poor	[89]
cyclin E1	52	mixed	yes	mixed	all	all	NLC-cyclinE (Novocastra)	retrospective	10 years			no	[41]
cyclin E1	53	mixed	yes	platinum-based	II-III	all	13A3 (Novocastra)	retrospective	-			poor	[57]
cyclin E1	134	serous	-	-	85% III-IV	all	HE12 (Pharmingen)	retrospective	29.8 months		no	no	[64]
cyclin E1	139	mixed	yes	cisplatin-based	III-IV	all	HE111 (Santa-Cruz)	prospective	-			poor	[60]
cyclin E1	180	serous	yes	platinum-based	all	all	polyclonal (Santa-Cruz)	retrospectivetiv e	18 months minimum		poor	poor	[248]
cyclin E1	405	78% serous	yes	cisplatin-based	all	90% G3	HE12 (Santa-Cruz)	retrospective	64 months			poor	[61]

Table 1. *Cont.*

DcR1	179	serous	yes	platinum-base	all	all	polyclonal	retrospective	31 months			no	[58]
DcR2	179	serous	yes	platinum-base	all	all	polyclonal	retrospective	31 months			no	[58]
Dr4	53	mixed	-	mixed	all	all	polyclonal (Santa-Cruz)	retrospective	63 months			no	[93]
Dr4	141	mixed	no	platinum-based	all	all	polyclonal C20 (Santa-Cruz)	prospective	29 months		no	no	[94]
Dr4	179	serous	yes	platinum-base	all	all	polyclonal	retrospective	31 months			no	[58]
Dr4	53	mixed	-	mixed	all	all	K-18 (Santa-Cruz)	retrospective	63 months			no	[93]
Dr4	141	mixed	no	platinum-based	all	all	PC392	prospective	29 months		no	no	[94]
Dr4	179	serous	yes	platinum-base	92% III-IV	all	polyclonal	retrospective	31 months			no	[58]
Dr4	75	all	no	mixed	II-IV	all	DJRI (Biolegends)	retrospective	23 months	poor CR	poor	poor	[97]
Dr5	75	all	no	mixed	II-IV	all	DJR2-4 (Biolegends)	retrospective	23 months	poor CR	no	no	[97]
E-cadherin	20	na	na	na	na	na	na	na	na			good	[249]
E-cadherin	72	serous	yes	mixed	all	all	NCH-38 (Dako)	retrospective	-			good	[222]
E-cadherin	95	serous	-	-	all	all	36B5 (DinonA)	retrospective	32 months			good	[221]
E-cadherin	104	mixed	yes	-	all	all	C2080 (Transduction Laboratories)	retrospective	-			good	[220]
E-cadherin	295	mixed	-	mixed	all	all	HECD-1 (Zymed)	retrospective	28 months	good		no	[223]
E-cadherin	45effusions	mixed	no	cisplatin/cyclophosphamide	III-IV	all	(Zymed)	retrospective	70 months			no	[241]
EGFR	18	serous	yes	platinum-based	III	all	EGFR.25 (Novocastra)	retrospective	-			no	[247]
EGFR	26	mixed	no	gefitinib	all	all	monoclonal (Zymed)	prospective	na		worse	poor	[250]
EGFR	28	endometrioid	na	na	all	all	na	na	na			poor	[205]
EGFR	41	mixed	yes	platinum-based	all	all	clone EGFR.25 (Novocastra)	retrospective	-			poor	[247]
EGFR	60	serous	no	cisplatin-based	III	G3	31G7 (Vantana)	retrospective	2 years		no	no	[153]
EGFR	75	mixed	-	-	all	all	monoclonal (Dako)	prospective	31 months			no	[216]
EGFR	77	mixed	-	mixed	all	all	-	retrospective	19 months		no	no	[251]
EGFR	84	mixed	yes	cisplatin/epirubicin/cyclophosphamide	all	all	EGFR113 (Novocastra)	retrospective	69 months		no	no	[43]
EGFR	90	mixed	-	paclitaxel/platinum	all	all	NCL-EGFR	retrospective	5-y	no	worse	poor (5-y)	[252]
EGFR	106	mixed	-	radiation	I-II	all	mouse (Novocastra)	retrospective	87 months			poor	[207]
EGFR	108	na	-	platinum-based	all	all	NA	retrospective	mean 50.7 months		worse	poor	[253]

Table 1. *Cont.*

EGFR	117	mixed	-	cisplatin-based	all	all	NA	retrospective	19 months			poor	[254]
EGFR	118	mixed	-	platinum-based	all	all	Clone H11 (Dako)	retrospective	72 months		no	no	[219]
EGFR	172	mixed	yes	-	all	all	Clone E30 (Dako)	retrospective	5-year			no	[255]
EGFR	230	mixed	-	cyclophosphamide/platinum	all	all	NCL-EGFR	retrospective	5-y	no	worse	poor (5-y)	[252]
EGFR	232	mixed	yes	platinum-based	all	all	31G7	prospective	-		no	no	[218]
EGFR	783	mixed	-	cyclophosphamide-based	all	all	EGFR113 (Novocastra)	prospective	214 months			no	[256]
EGFRnuclear	187	-	-	-	-	-	monoclonal (Novocastra)	retrospective	51.4 months			poor	[257]
EphB1	112	mixed	-	-	all	all	C-18 (Santa-Cruz)	prospective	31 months			no	[216]
EphB4	72	mixed	yes	-	all	all	polyclonal (Santa-Cruz)	retrospective	-			poor (2yrs)	[211]
EphB4	72	mixed	-	platinum-based	III-IV	all	Polyclonal (Santa-Cruz)		71 months	poor		poor	[210]
EphB4	85	mixed	-	-	all	all	Mab131 (Vasgene therapeutics)		-			poor	[209]
ErbB3	28	endometrioid	na	na	all	all	na	na	na			poor	[205]
ErbB3	116	mixed	-	platinum-based	all	all	C-17 (Santa Cruz)	retrospective	-			poor	[206]
ERCC-1	63	serous	-	platinum-based	III-IV	all	monoclonal	retrospective	-	poor			[258]
ERCC-1	101	mixed (84% serous)	yes	platinum-based	all	all	clone 8F1 (Neomarker)	retrospective	19 months	poor	poor	poor (p=0.10)	[121]
ERCC-1	59	serous	-	platinum-based	II-III	all	8F1 (Neomarker)	retrospective	minimum 6 months	no			[259]
Fas	35	mixed	no	mixed	all	all	UB-2 (MBL)	retrospective	32 months			no	[96]
Fas	75	80% serous	no	mixed	all	all	-	retrospective	23 months	poor CR	no	no	[97]
Fas	141	mixed	no	platinum-based	all	all	CH-11 (Upstate)	prospective	29 months		good	good	[94]
Fas-L	35	mixed	no	mixed	all	all	clone 33 (Transduction Laboratories)	retrospective	32 months			poor	[96]
FGF-2 /bFGF	50	mixed	-	platinum-based and adriamycin/cyclophosphamide	all	all	R&D system	retrospective	42 months			good	[260]
FGF-2 /bFGF	67	mixed	yes	mixed platinum-based	III-IV	all	F6162 (Sigma)	retrospective	67 months		good	good	[159]
FLIP	141	mixed	no	platinum-based	all	all	NF-6	prospective	29 months		no	no	[94]
FLIP	179	serous	yes	platinum-base	all	all	clone G-11	retrospective	31 months		no		[58]
FLIP	207	mixed	na	na	na	na	na	na	na			poor with p53	[95]
FLIP(L)	54	mixed	-	mixed	all	all	polyclonal C-19 (Santa-Cruz)	retrospective	63 months			no	[93]

Table 1. *Cont.*

Her-2	43	mixed	yes	cisplatin-based	III	all	na	retrospective	-			no	[175]
Her-2	44	serous	na	na	na	na	HercepTest	na	na			no	[261]
Her-2	52	na	na	na	III	na	HercepTest	na	66 months			no	[36]
Her-2	72	mixed	-	85% paclitaxel	-	-	HercepTest	retrospective	33 months		no	no	[34]
Her-2	75	mixed	-	-	all	all	monoclonal (Biogenex)	prospective	31 months			no	[262]
Her-2	95	mixed	-	paclitaxel/carboplatin	all	all	MC0102 (Ylem)	prospective	66 months	no	no	no	[106]
Her-2	100	mixed		Cyclophosphamide/platinum or paclitaxel/platinum	III-IV	na	na	prospective	55 months (median)	no	no	no	[239]
Her-2	105	mixed	na	na	III-IV	all	na	retrospective	34 months			no	[263]
Her-2	106	mixed	-	radiation	I-II	all	polyclonal (Dako)	retrospective	87 months			no	[207]
Her-2	116	mixed	-	platinum-based	all	all	HercepTest	retrospective	39 months			no	[108]
Her-2	117	mixed	-	cyclophosphamide	III-IV	all	CB11(Novocastra)	retrospective	68 months	no	poor	poor	[44]
Her-2	118	mixed	-	platinum-based	II-IV	all	CB11(Novocastra)	retrospective	72 months		poor	trend	[219]
Her-2	128	mixed	-	mixed	all	all	HercepTest	retrospective	44 months			poor	[264]
Her-2	130	mixed	-	-	III-IV	all	HercepTest	retrospective	-			poor	[265]
Her-2	141	mixed	-	platinum-based	all	all	polyclonal	retrospective	-	no		no	[266]
Her-2	160	mixed	-	carboplatin-based	all	all	HercepTest	retrospective	at least 10 years			poor	[178]
Her-2	181	mixed	-	-	all	all	HercepTest	retrospective	52 months			poor	[265]
Her-2	194	mixed	-	platinum-based	all	all	HercepTest	retrospective	45 months		poor	poor	[267]
Her-2	232	mixed	yes	mixed	all	all	NCL-CBE-356	prospective	-		no	no	[218]
Her-2	320	mixed	-	carboplatin-based	all	all	CB11(Novocastra)	prospective	25 months		no	no	[268]
Her-2	783	mixed	-	cyclophosphamide-based	all	all	polyclonal (Dako)	prospective	214 months			good	[256]
Her-2	783	mixed	-	mixed	II-IV	all	HercepTest	retrospective	57 months	no	no	no	[269]
Her-2	45	mixed	-	cisplatin/cyclophosphamide	III-IV	all	Novocastra	retrospective	70 months			poor	[241]
Her-2	73	mixed	no	-	III-IV	all	TA1 (Applied Technology Inc.)	retrospective	-			poor	[270]
Her-2	77	mixed	-	cisplatin or carboplatin	all	all	Dianon system	retrospective	19 months		no	no	[251]
HIF-1□	52	mixed	-	paclitaxel/carboplatin	III-IV	all	Novus Biologicals	retrospective	>48 months			good	[167]
HIF-1□	55	serous	-	mixed	III	G3	HIF a67, 54/HIF, polyclonal	retrospective	3 years (mean)		poor	poor	[164]
HIF-1□	72	mixed	-	cisplatin-based	all	all	ESEE122 (Novus Biologicals)	retrospective	52 months			poor	[166]
HIF-1a	112	serous	-	mixed	all	all	ESEE122 (Turley)	retrospective	-			poor	[152]
HIF-1□	102	mixed	-	platinum/taxol	all	all	HIFa67 (Novus Biologicals)	retrospective	28 months			no	[149]
Hsp 27	52	mixed	yes	-	all	all	G3.1 (Neomarker)	retrospective	32 months			poor	[271]
Hsp 70	52	mixed	yes	-	all	all	W27 (Neomarker)	retrospective	32 months			no	[271]

Table 1. *Cont.*

Hsp 90	52	mixed	yes	-	all	all	polyclonal (Neomarker)	retrospective	32 months			no	[271]
Hsp 27	157 effusion	mixed	no	-	III-IV	all	-	prospective	26 months			no	[113]
Hsp 70	157 effusion	mixed	yes	-	III-IV	all	-	prospective	26 months			poor	[113]
Hsp 70	65 effusion	mixed	post	-	III-IV	all	-	prospective	26 months			poor	[113]
Ki67	30	na	na	cisplatin/paclitaxel	na		na	na	na			no	[272]
Ki67	36	mixed	-	platinum	III-IV	72% gr3	MIB-1	retrospective	-			poor	[39]
ki67	51	mixed	-	platinum	III	all	MIB-1	retrospective	-			poor	[273]
Ki67	52	mixed	yes	mixed	all 50%	all	MIB-1	retrospective	10 years			poor	[41]
ki67	52	na	na	na	III	na	na	na	66 months			poor	[36]
Ki67	54	serous	-	mixed	more high stage	all	MIB-1	retrospective	34 months		poor		[26]
Ki67	66	mixed	yes	platinum-based	all	all	MIB-1	retrospective	25 months		poor	poor	[35]
ki67	69	mixed	yes	-	all	all	monoclonla (Immunotech)	retrospective	30 months			no trend	[15]
Ki67	72	all	-	85% paclitaxel		all	-	retrospective	33 months		no	no	[34]
ki67	77	mixed	-	cisplatin or carboplatin	all	all	monoclonal	retrospective	19 months		poor	poor	[251]
Ki67	77	61% serous	-	65% platinum	75% III-IV	high	MIB-1	retrospective	14 months			no	[30]
ki67	80	mixed	yes	mixed	all	all	MIB-1	retrospective	50 months			poor	[32]
Ki67	90	mixed	-	-	all	all	MIB-1	-	-		poor	poor	[56]
ki67	93	mixed	yes	cisplatin/epirubicin/cyclophosphamide	all	all	MIB-1	retrospective	69 months		no	no	[43]
ki67	105	mixed	-	platinum/cyclophosphamide	III-IV	-	MIB-1	retrospective	minimum 5 years			poor	[33]
ki67	116	mixed	-	platinum-based	all	all	MIB-1	retrospective	39 months			poor	[108]
ki67	117	mixed	yes	cisplatin/epirubicin/cyclophosphamide	all	all	MIB-1	retrospective	68 months		no	no	[44]
ki67	118	serous	yes	-	III-IV	G3	MM1(Vector laboratory)	retrospective	1.7 years			poor	[150]
ki67	118	serous	yes	-	III-IV	high	K-2 (Ventana)	retrospective	1.36 years			poor	[42]
Ki67	132	clear cells	-	platinum-based	all	all	SP-6 (Labvision)	retrospective	6.3 yrs (mean)			no	[16]
ki67	134	94% serous	-	platinum-based	III-IV	high	DAKO	retrospective	42 months (mean)			poor (5 yrs)	[38]
ki67	169	mixed	-	cyclophosphamide/carboplatin	all	all	MIB-1	retrospective	10 years		yes	no	[45]
Ki67	171	mixed	-	cisplatin/epirubicin	III	all	KI-S5 (Dako)	retrospective	yes	no	poor	poor	[22]

Table 1. *Cont.*

Ki67	200	serous	-	platinum-based	all	high	SP-6 (Labvision)	retrospective	5.4 yrs (mean)			no	[16]
ki67	204	mixed	-	cyclophosphamide/cisplatin-based	II-IV	all	MIB-1	retrospective	35.2 months		poor	no	[78]
ki67	251	mixed	na	na	all	all	MIB-1	retrospective	na			poor	[40]
ki67	500	mixed	-	platinum-based	all	all	SP-6 (Labvision)	retrospective	5.1 yrs			poor	[16]
Mcl-1	185	mixed	yes	cisplatin/epirubicin	III	all	polyclonal	retrospective	85 months		poor	poor	[77]
NF-kB(p65)	63	mixed	yes	-	all	all	monoclonal (Santa Cruz)	retrospective	30 months			poor	[214]
NF-kB (p65)	166	serous	no	mixed	III-IV	all	polyclonal (Abcam)	retrospective	31 months		poor		[117]
NF-kB(p65)	166	serous	-	mixed	all	all	polyclonal (Abcam)	retrospective	31 months	poor		poor	[117]
p16	35	mixed	-	platinum paclitaxel	III	all	mouse (Chemicon Int.)	retrospective	yes		no	no	[88]
p16	43	mixed	-	mixed platinum-based	all	all	mouse (Chemicon Int.)	retrospective	yes	good		good	[88]
p16	47	mixed	yes	-	all	all	F12 (Santa-Cruz)	retrospective	-			no	[89]
p16	52	mixed	-	-	all	all	16PO4 (Neomarker)	retrospective	6months-11 yr			poor	[90]
p16	59	mixed	-	cyclophosphamide/adriamycin/ platinum	all	all	-	-	22 months			no	[65]
p16	77	mixed	-	65% platinum	all	high	G175-405	retrospective	14 months			no	[30]
p16	134	serous	-	-	all	all	16PO4 (Neomarker)	retrospective	29.8 months		no	no	[64]
p16	159	mixed	-	-	all	all	-	retrospective	mini 3yrs			poor	[91]
p16	159	mixed	-	-	all	all	-	retrospective	mini 3yrs			good	[91]
p16	171	74% serous	yes	cisplatin/epirubicin	III	all	16PO4 (Neomarker)	retrospective	-	no		poor	[22]
p16	300	mixed		platinum/paclitaxel	II-IV	-	E6H4 (mtm laboratories)	retrospective	-			good	[87]
p21	43	85%serous	yes	platinum-based	III	all	SX118 (dako)	retrospective	-		good	good	[82]
p21	52	mixed	yes	mixed	all	all	Ab-1 (Oncogen Science)	retrospective	minimum 10 years			no	[41]
p21	66	mixed	yes	platinum-based	all	all	Waf1-EA10 (Oncogene Research)	retrospective	25 months	no	no	no	[35]
p21	69	mixed	no	mixed	all	all	2G12 (Pharmingen)	retrospective	78 months	no	no	no	[85]
p21	77	mixed	yes	platinum-based	I	all	Dako	retrospective	73 months		no	no	[142]
p21	77	mixed	-	65% platinum	all	high	6B6 (Pharmingen)	retrospective	14 months			good	[30]
p21	77	mixed	-	65% platinum	all	high	1B4(Novocastra)	retrospective	14 months			no	[30]
p21	102	mixed	na	na	III-IV	na	na	retrospective	na		good (3 yr)	good	[81]
p21	106	mixed	-	-	all	all	Waf1(Oncogene science)	retrospective	32 months		no	good	[83]

Table 1. *Cont.*

p21	120	mixed	yes	mixed	all	all	Waf1 (Oncogene science)	retrospective	24 months	no	no	no	[84]
p21	134	serous	-	-	85% III-IV	all	clone 70	retrospective	29.8 months		good	good	[64]
p21	169	mixed	-	cyclophosphamide/cisplatinum	II-III-IV	all	2G12 (Pharmingen)	retrospective	9 years		good	good	[45]
p21	185	mixed		cisplatin/epirubicin	III	all	Ab-1 (Oncogen Science)	retrospective	77 months			no	[104]
p21	200	serous	-	94% platinum-based	all	high	DCS-60.2Llabvision)	retrospective	5 yrs			no	[16]
p21	204	mixed	-	platinum-based	all	all	sc-6246 (Santa-Cruz)	retrospective	35.2 months	good	good	good	[78]
p21	267	mixed	-	platinum-based	all	all	Dako	retrospective	3.9 years			good	[274]
p21	500	mixed	-	94% platinum-based	all	all	DCS-60.2 (Labvision)	retrospective	5yrs			no	[16]
p27	47	mixed	yes	platinum-based	all	all	monoclonal (Transduction laboratories)	retrospective	24 months			good	[59]
p27	52	mixed	yes	mixed	all	all	NCL-p27 (Novocastra)	retrospective	minimum 10 years			good	[41]
p27	53	mixed	yes	platinum-based	II-III	all	K25020	retrospective	minimum 2 years			good	[75]
p27	72	mixed	yes	mixed	all	all	1B4(Novocastra)	retrospective	50 months			good	[32]
p27	75	75% serous	yes	platinum-based (100%) with taxane (83%)	III-IV	high	monoclonal (Transduction Laboratories)	retrospective	28 months (mininmum 6months)	no	no	no	[62]
p27	99	80% serous	-	cisplatin	III-IV	all	DCS-72.F6 (Neomarker)	retrospective	27 months			good	[74]
p27	117	na	na	na	all	all	na	na	na			good	[73]
p27	134	serous	-	-	all	all	clone 57 (Tranduction Laboratory)	retrospective	29.8 months		good	good	[64]
p27	165	mixed	-	mixed	all	all	monoclonal (Transduction Laboratories)	retrospective	39 months	no	good	good	[76]
p27	185	mixed	-	cisplatin/epirubicin	III	all	K25020 (Transduction Laboratories)	retrospective	77 months			no	[104]
p27	204	mixed	-	platinum-based	all	all	NCL-p27	retrospective	35.2 months	no			
p27	305	mixed	-	mixed	all	all	M7203 (Dako)	prospective	-			poor	[275]
p27	421	mixed	na	na	na	na	na	na	na			poor	[79]
p53	43	na	na	paclitaxel-based	II-IV	na	na	retrospective	na		no	no	[276]
p53	43	mixed	yes	platinum-based	III	all	DO-7	retrospective	-		poor	poor	[82]
p53	49	mixed	-	-	all	all	DO-7	retrospective	-		no	no	[110]

Table 1. *Cont.*

p53	52	mixed	yes	mixed	all	all	DO-1	retrospective	minimum 10 years			no	[41]
p53	55	mixed	yes	cisplatin/ doxorubicin	all	all	pAb1801 (Novocastra)	retrospective	38 months			poor	[277]
p53	60	serous	yes	paclitaxel/ carboplatin	all	all	BP53.12 (Zymed)	retrospective	42 months	no		no	[238]
p53	66	mixed	yes	platinum-based	all	all	DO-1	retrospective	25 months		poor	poor	[35]
p53	70	mixed	-	platinum-based	all	all	DO-7	retrospective	7 years		no	poor	[109]
p53	72	all	-	85% paclitaxel	all	all	-	retrospective	33 months		no	no	[34]
p53	77	mixed	yes	platinum-based	I	all	na	retrospective	73 months		poor	no	[142]
p53	79	mixed	no	platinum-based	all	all	DO-1	retrospective	78 months		trend	poor	[85]
p53	80	mixed	yes	mixed	all	all	DO-1	retrospective	50 months			no	[32]
p53	90	mixed	-	mixed	all	all	DO-7	retrospective	-		no	no	[112]
p53	90	mixed	-	-	all	all	DO-1	-	-		no	no	[56]
p53	90	mixed	na	na	all	all	na	retrospective	na			poor	[278]
p53	93	mixed	yes	cisplatin/epirubicin/cy clophosphamide	all	all	DO-7	retrospective	69 months		no	no	[43]
p53	95	mixed	-	paclitaxel/ carboplatin	all	all	DO-1	prospective	66 months	no	no	good	[106]
P53	100	mixed		cyclophosphamide/pla tinum or paclitaxel/platinum	III-IV	na	na	prospective	55 months (median)	no	no	no	[239]
p53	103	mixed	-	platinum-based	all	all	pAb1801 (Novocastra)	retrospective	60 months			poor	[80]
p53	105	mixed	-	cyclophosphamide/do xorubicin/ cisplatin	all	all	DO-7	retrospective	minimum 1 month	no			[103]
p53	106	mixed	-	radiation	I-II	all	DO-7	retrospective	87 months			poor	[99]
p53	107	mixed	-	mixed	all	all	Dako-p53	retrospective	7 yrs			poor	[279]
p53	116	mixed	-	platinum-based	all	all	DO-1	retrospective	39 months			no	[108]
p53	118	serous	yes	-	III-IV	high	DO-7	retrospective	1.36 years			no	[42]
p53	119	serous	-	platinum-based	all	all	DO-7	retrospective	46.4 months			no	[280]
p53	134	serous	-	mixed	all	all	DO-7	retrospective	29.8 months		poor	poor	[64]
p53	136	mixed	na	na	all	all	na	na	10 years			poor	[281]
p53	140	mixed		palitacxel/cisplatin	all	all	2D2	retrospective	31 months			poor	[98]
p53	151	mixed		platinum/taxane	all	all	DO-7	retrospective	-		no	no	[165]
p53	164	mixed	yes	cisplatin/epirubicin/cy clophosphamide	all	all	DO-7	retrospective	68 months		no	no	[44]
p53	169	mixed		cyclophosphamide/cis platinum	all	all	DO-1	retrospective	9 years		no	no	[45]
p53	171	mixed	-	mixed	all	all	DO-7	retrospective	3.4 yrs		no	no	[282]
p53	178	mixed	-	platinum-based	all	all	DO-7	retrospective	31 months		poor	poor	[283]
p53	199	mixed	yes	taxane/platinum	all	all	B-9 (Santa Cruz)	retrospective	29 months				[102]
p53	251	mixed	na	na	all	all	na	retrospective	na			poor	[40]

Table 1. *Cont.*

p53	284	mixed	na	na	all	all	na	na	na			poor	[284]
p53	500	mixed	-	platinum-based	all	all	DO-7	retrospective	5.1 years			no	[16]
p53	783	mixed	-	cyclophosphamide-based	all	all	DO-7	prospective	214 years			poor	[256]
p57	47	mixed	yes	platinum-based	all	all	monoclonal (Transduction laboratories)	retrospective	24 months			good	[89]
p57	53	mixed	yes	platinum	II-III	all	polyclonal	retrospective	-			no	[57]
p57	171	all	yes	cisplatin/epirubicin	III	all	57PO6	retrospective	-	no		no	[22]
PCNA	28	na	na	platinum-based	all	all	na	retrospective	na			poor	[285]
PCNA	49	na	na	na	na	na	PC-10 (Dako)	na	na			trend	[28]
PCNA	92	na	yes	platinum-based	III-IV	na	na	na	na			good	[49]
PCNA	67	mixed		mixed	all	all	PC-10 (Dako)	retrospective	60 months		no	no	[46]
survivin	49	mixed	-	-	all	all	polclonal (Santa-Cruz)	retrospective	-		no	no	[110]
survivin	100	mixed	yes	platinum-based	all	all	clone A-b-6 (Neomarker)		67 months			poor	[173]
survivin	101	mixed	yes	platinum-based	all	all	polyclonal (Novus)	retrospective	20 months		good	good	[118]
survivin	47	mixed		platinum-based	all	all	na	retrospective	24 months			poor	[86]
survivin	110	mixed		platinum-based	all	all	ab496 (Abcam)	retrospective	32 months		no	no	[286]
THBS-1	33	mixed	-	platinum-based	III	G2-3	Mob 315 (Dako)	retrospective	37 months			no	[180]
THBS-1	36	mixed		mixed	I-II	all	monoclonal (Neomarker)	retrospective	9 years			no	[179]
THBS-1	49	mixed	no	mixed	all	all	monoclonal (Neomarker)	retrospective	9 years			trend	[179]
THBS-1	67	mixed	yes	mixed platinum-based	III-IV	all	T2905 (Sigma)	retrospective	67 months		poor	poor	[159]
THBS-1	77	mixed	yes	platinum-based	I	all	Oncotech	retrospective	73 months		no	no	[142]
TNFR1	75	mixed	no	mixed	II-IV	all	-	retrospective	23 months	no	no	no	[97]
TNFR2	75	mixed	no	mixed	II-IV	all	-	retrospective	23 months	no	no	no	[97]
topoIIa	37	na	yes	cisplatin	na	na	na	na	na				[287]
topoIIa	96	mixed	-	platinum-based	III-IV	all	Ki-S1 (Dako)	retrospective	37 months		no	poor	[54]
topoIIa	36	mixed	-	platinum	III-IV	all	SWt3D1(Antibody online)	retrospective	-			poor	[39]
topoIIa	41	serous	yes	platinum	all	all	Ki-S1 (Dako)	retrospective	-			poor	[53]
topoIIa	90	mixed	-	-	all	all	SWt3D1(AntibodyOnline)	-	-		no	no	[56]
topoIIa	133	mixed	yes	caboplatin/ paclitaxel	all	all	polyclonal (BioTrend Chemicals)	retrospective	-		poor	poor	[51]
topoIIa	108	mixed	-	platinum-based	all	all	-	-	54 months			poor	[288]
TRAIL	37	mixed		mixed	III-IV	all	K-18 (Santa-Cruz)	retrospective	-			good	[93]
TRAIL	53	mixed		mixed	all	all	K-18 (Santa-Cruz)	retrospective	63 months			no	[93]
TRAIL	141	mixed	no	platinum-based	all	all	K-18 (Santa-Cruz)	prospective	29 months			good	[94]
TRAIL	179	serous	yes	platinum-based	all	all	polyclonal	retrospective	31 months		no	good	[58]

Table 1. *Cont.*

VEGF	33	mixed	-	platinum-based	III	G2-3	JH121 (Neomarker)	retrospective	37 months			no	[180]
VEGF	32	serous		cisplatin/epirubicin/cyclophosphamide	all	all	Polyclonal (Santa-Cruz)	retrospective	minimum 3 years			poor	[158]
VEGF	39	mixed	-	platinum-based	all	all	-	retrospective	24 months		poor	poor	[157]
VEGF	45	mixed	na	platinum-based	III-IV	all	na	retrospective	na	no	no		[160]
VEGF	45	mixed	-	cisplatin, adriamycin/cyclophosphamide	I-II	all	polyclonal (Santa-Cruz)	retrospective	-		good		[143]
VEGF	60	mixed	-	cisplatin, adriamycin/cyclophosphamide	III-IV	all	polyclonal (Santa-Cruz)		-		no		[143]
VEGF	60	serous	no	cisplatin-based	III	G3	monoclonal (Santa-cruz)	retrospective	-		poor	poor	[153]
VEGF	64	mixed	-	mixed	all	all	polyclonal (Santa-Cruz)	retrospective	31 months			poor	[154]
VEGF	66	na	na	na	all	na	na	na	na		poor	poor	[155]
VEGF	67	mixed	yes	mixed platinum-based	III-IV	all	V6627 (Sigma)	retrospective	67 months		no	no	[159]
VEGF	70	mixed	-	cisplatin-based	all	all	polyclonal (Santa-Cruz)	retrospective	-			poor	[156]
VEGF	77	mixed	yes	platinum-based	I	all	Neomarker	retrospective	73 months		poor	poor	[142]
VEGF	79	mixed	-	platinum-based	all	all	clone 14-12 (Oncogene)	retrospective	-		poor	poor	[162]
VEGF	83	serous	-	platinum-based	III	G3	monoclonal (Santa-Cruz)	retrospective	45 months		poor	poor	[245]
VEGF	112	serous	-	mixed	all	all	VG1 (DCS)	-	-		good	no	[152]
VEGF	320	mixed	yes	platinum-based	all	all	polyclonal (Abcam SP28)	retrospective	-			poor	[161]
VEGF	314	mixed		platinum-based	all	all	na	retrospective	39 months			poor	[163]
WT-1	69	mixed	yes	-	all	all	polyclonal (Santa-Cruz)	retrospective	30 months			no	[15]
WT-1	99	serous	-	platinum/ cyclophosphamide	III-IV		monoclonal	retrospective	-			poor	[19]
WT-1	100	mixed	-	-	all	all	6F-H2(Dako)	retrospective	30 months		no	no	[14]
WT-1	119	serous	-	platinum-based	all	all	6F-H2	retrospective	46 months			poor	[20]
WT-1	200	serous	-	platinum-based	all	high	6F-H2	retrospective	5yrs			good	[16]
WT-1	500	mixed	-	platinum-based	all	all	6F-H2	retrospective	5.1 years			poor	[16]
WT-1	560	mixed	-	mixed	all	all	6F-H2	retrospective	23 months			poor	[18]
XIAP	101	mixed	yes	platinum-based	all	all	clone 48 BDsciences	retrospective	20 months		no	no	[118]

* in TP53 mutant patients. CN= Chemonaive patients. CR= chemotherapy response. DFS=disease free survival. OS=overall survival. Na+information non available.
